# Intraspecific anatomical variations of the extensor tendons of the carpus and digits with a reexamination of their insertion sites in the domestic dog (*Canis lupus familiaris*): a cadaveric study

**DOI:** 10.1186/s12917-023-03750-w

**Published:** 2023-10-09

**Authors:** Younes Kamali, Reyhaneh Almasi, Hamid Reza Moradi, Saeid Fathollahi

**Affiliations:** Department of Basic Sciences, School of Veterinary Medicine, Shiraz University, Shiraz, Iran

**Keywords:** Extensor tendon variants, Manus, Frequency, Brachioradialis muscle, Mixed-breed dogs

## Abstract

**Background:**

The aim of the current study was to investigate the frequency of variations of the extensor tendons of the carpus and digits in the domestic dog (*Canis lupus familiaris*) with a reexamination of their insertions as well as the morphometric measurements of the tendons and the brachioradialis muscle. In total, we investigated 68 paired thoracic limbs of the domestic dog (16 females and 18 males) which were fixed in a 10% formalin solution.

**Results:**

The extensor carpi radialis (ECR) tendons showed striking variations in both splitting and insertion sites. In 4.4% of dissections, ECR had three tendons. Of these tendons, the extra tendon either attached independently on the fourth metacarpal bone (one right) or joined its counterpart tendon at the distal end (cross-connections) (one bilateral). It is worth mentioning that one of the ECR tendons split into two or three slips which inserted on the first, second, third, or fourth metacarpal bone in 11 (16.2%) of the specimens. In addition, we found a long tendinous slip originating from the ECR tendons to digit II or III in 7.4% of the distal limbs. The most common type of contribution to digit III was a third tendon of the extensor digiti I et II (ED III) joining the extensor digitorum lateralis (EDL III) with a frequency of 17.6%. In other types of variations, the contribution to digit III was incomplete. A part of the abductor pollicis longus (APL) deep to the superficial part of the flexor retinaculum seemed to continue up to the flexor digitorum superficialis (FDS) tendon.

**Conclusions:**

The rare intraspecific variations of the extensor tendons of the manus described in the current research are valuable from both clinical and phylogenetic perspectives. Nonetheless, their functional importance needs more studies.

**Supplementary Information:**

The online version contains supplementary material available at 10.1186/s12917-023-03750-w.

## Background

Cursorial animals often show an evolutionary elongation of distal limb proportions and a reduced number of digits. In canids, for example, digit I (dew claw) tends to be lost or reduced, leaving the four well-developed ones (II to V) to bear the weight of the body [1]. Another typical adaptation in quadrupedal cursors is an increase in the length and number of tendons in the manus [2]. Similarly, cursorial carnivorans which do not need dexterous movements to manipulate their prey have a less evolved muscle mass in the distal parts of the thoracic limb [3]. Hence, the anatomy of the thoracic limb muscles in the order Carnivora has been a great research interest regarding relevant phylogenetic adaptations. Moreover, among carnivores, a marked interspecific variation exists in the muscles and tendons of the antebrachium, particularly in those of the craniolateral compartment [4, 5].

For descriptive purposes, the muscles of the antebrachium in the domestic dog (*Canis lupus familiaris*) are divided into two functional compartments (i.e. craniolateral and caudomedial). The craniolateral antebrachial muscles include the brachioradialis (BR), abductor digiti I (pollicis) longus (APL), extensor carpi radialis (ECR), extensor digiti I et II (ED I et II), extensor digitorum communis (EDC), extensor digitorum lateralis (EDL), and extensor carpi ulnaris (ECU) [6, 7]. Functionally, except for the BR and ECU, these muscles mainly extend the carpus. Additionally, they extend the digits based on the attachment sites of their insertion tendons [8]. The ECR is represented by a partial division of the muscle belly and two definite tendons inserting into the bases of metacarpal bone II (extensor carpi radialis longus) and metacarpal bone III (extensor carpi radialis brevis) [6, 7, 9, 10]. On their course to their insertion on the extensor expansion of the distal phalanx of digits II to V, the four EDC tendons receive contributions from the three EDL tendons to the lateral digits and the second tendon from ED I et II to digit II, or rarely from an additional tendon from the latter to digit III [7].

In humans, during phylogenesis, the extensor tendons of the antebrachium and manus have evolved in a more complex pattern to adapt specifically to various types of movements. Hence, the arrangement and anatomic variations of the extensor tendons of the carpus or digits as well as their prevalence have been discussed in several excellent studies [11–15]. In addition, there are oblique interconnections, termed juncturae tendinum (connexus intertendinei), between the two adjacent extensor digitorum tendons distally, except between the first and second ones on the dorsum of the manus [11]. Whether the homology of these structures is seen in domestic dogs or not has not been previously recorded in veterinary anatomical texts.

Published data on thoracic limb muscle morphology are available for a wide variety of wild carnivorans. However, most of these studies have examined only a very limited number of wild carnivorans partly because they are endangered species and partly because they are difficult to find. This has prevented the identification of more rare variations for relevant phylogenetic analyses [3]. Even though the morphology of the craniolateral antebrachial muscles in domestic dogs has been described and illustrated [6, 7, 10], the variability and frequency of the tendons have rarely been taken into consideration. Some variations have been briefly mentioned in very old texts without providing any photographs [4, 16]. Diogo et al. are the authors who have put as much emphasis on the reliability of muscle characteristics as on osteological characteristics in the phylogenetic studies of higher-level clades of mammals [17]. Paying attention to muscle and tendon variations as well as considering the frequency of occurrence in a larger carnivoran population with easier accessibility such as domestic dogs can be helpful in revealing the phylogenetic relationships among carnivorans. In addition, they play an important role in the phylogenetic reconstructions of other wild taxa [18, 19]. Comparing these variants with those occurring in other carnivores or even in their common ancestors is useful in showing which ones are new and have never occurred before in the evolution, and conversely, which ones are reversal, vestigial, or atavistic variants [20].

From the clinical standpoint, the domestic dog which has five digits could be given more attention in medical research as a model for advancing surgical goals for tendon repair [21]. Detailed anatomical knowledge of extensor tendons is increasingly needed in veterinary medicine for assessing the tendons with new diagnostic imaging modalities (such as ultrasonography) or for planning surgical interventions for tendon injuries in dogs participating in various sports [22, 23]. Although the rupture of the extensor tendons in the distal limbs of small animals does not have serious clinical consequences in contrast to that of the flexor ones [24], thanks to surgical advances, the variants of these tendons as extra tendons or slips (depending on their sizes) can be used for tendon transfer and reconstruction of other more important tendons as suggested in medical texts [25]. Moreover, the awareness of tendon variations and their frequency can prevent sudden tendon severance during surgical incision on the dorsal aspect of the manus and reduce the possibility of false diagnoses in imaging. For instance, the additional attachment sites of the split ECR tendons may lead to the development of new avulsion fractures on the bases of the other metacarpal bones [26, 27].

The aims of the present study were to investigate for the first time the frequency of the intraspecific variations of the extensor tendons of the carpus and digits from their origin at the myotendinous junction (MTJ) to insertion, to obtain the sizes of the tendons and the BR, and to describe the insertion sites of the tendons by considering their adjacent structures (such as the relationship of the APL tendon with the flexor retinaculum). Some possible functional and phylogenetic aspects of these variations are also discussed in this paper.

## Results

The common pattern of the extensor tendons on the dorsum of the manus was found in 38.2% of the limbs.

The variations and unusual tendons observed in this study (with some specimens showing two or more of these variations) can be classified into 7 groups as follows: [1] the three tendons of the ECR and the cross-connections; [2] the splitting of the ECRL or the ECRB into two or three slips; [3] a long tendinous slip from the ECRL or the ECRB to the digits; [4] the contribution of tendon(s) arising from the EDL and/or ED I et II to digit III; [5] the splitting of the second tendon of the ED I et II into two slips; [6] the splitting of the EDC tendon of digit III (EDC III); [7] a fascial to tendinous accessory slip stemming from the extensor tendons of the digits (Table 1).

**Table 1:** Intraspecific variations of the extensor tendons of the carpus and digits in the domestic dog

**Register number**	**Sex**	**Side**	**ECRL**	**ECRB**	**ED I et II**	**EDC**	**EDL**
1	F	R	-	A double tendon for the bases of metacarpals III and IV	Having ED III	-	-
		L	-	Having a fan-shaped tendinous expansion to insert on the shaft of metacarpal V	Having ED III	-	-
2	F	R	A double tendon; the extra one joining the main tendon of its counterpart near the distal end	Splitting above the carpus to insert on the bases of metacarpals III and IV	-	-	-
		L	A double tendon; the extra one joining the main tendon of its counterpart near the distal end	Splitting at the carpus to insert on the bases of metacarpals III and IV; having a long tendinous slip to unite with ED II	Anomalous origin of ED III	-	-
3	M	R	-	Splitting above the carpus; the abaxial slip inserting on the lateral aspect of the base of metacarpal III	-	-	-
		L	Splitting above the carpus; the abaxial slip inserting on the base of metacarpal III medial to the insertion of ECRB	-	-	-	-
4	M	R	-	-	-	-	-
		L	Having a thin tendinous slip to insert on the base of metacarpal I	-	-	-	-
5	M	R	Having a long tendinous slip to unite with EDC II at the MCP joint as well as a fine tendinous slip to insert on the medial aspect of the base of metacarpal II	Having a fan-shaped tendinous expansion to insert on the base of metacarpal IV	Ectopic insertion of ED II into digit III	-	-
		L	-	Having a long tendinous slip to unite with EDC III next to the insertion of EDL III	-	-	-
6	F	R	-	Having a fine tendinous slip to insert on the carpal joint capsule	-	-	-
		L	-	-	-	EDC III having a sagittal fissure at the MCP joint	-
7	F	R	-	Splitting at the distal end; the abaxial slip inserting on the bases of metacarpals III and IV	-	-	An absent EDL III
		L	-	Splitting at the distal end; the abaxial slip inserting on the bases of metacarpals III and IV	-	-	An absent EDL III replaced by a fascial band
8	M	R	-	Partial splitting at the carpus	-	-	-
		L	-	Partial splitting at the carpus	-	-	-
9	F	R	-	-	-	-	-
		L	-	Having a long tendinous slip to unite with EDL III	-	-	-
10	F	R	-	-	-	-	-
		L	-	Having a long tendinous slip to unite with EDL III	-	-	-
11	M (German Shepherd)	R	-	-	Having ED III	-	An absent EDL III
		L	-	-	Having a split ED II with two insertions; the anomalous origin of ED III	-	Having an accessory slip stemming from EDL V above the MCP joint
12	F	R	-	-	Having ED III	-	-
		L			Having a split ED II with one insertion; the replacement of ED III by a fascial band		
13	F	R	-	-	-	-	-
		L	-	-	-	-	Ectopic origin of EDL III from the dorsal metacarpal fascia
14	F	R			Having a split ED II with two insertions		
		L			Having a split ED II with two insertions		
15	M	R	-	-	Having ED III	An oblique connecting filamentous band between EDC IV and EDC V	-
		L	-	-	Having ED III	-	-
16	F	R	-	-	Having ED III	-	Having an accessory slip stemming from EDL V above the MCP joint
		L	-	-	-	-	Having an accessory slip stemming from EDL V above the MCP joint
17	M	R	-	-	-	EDC III having a sagittal fissure at the MCP joint	-
		L	-	-	-	-	-

### The three tendons of the ECR and the cross-connections (three distal limbs -4.4%-)

**Fig. 1:**
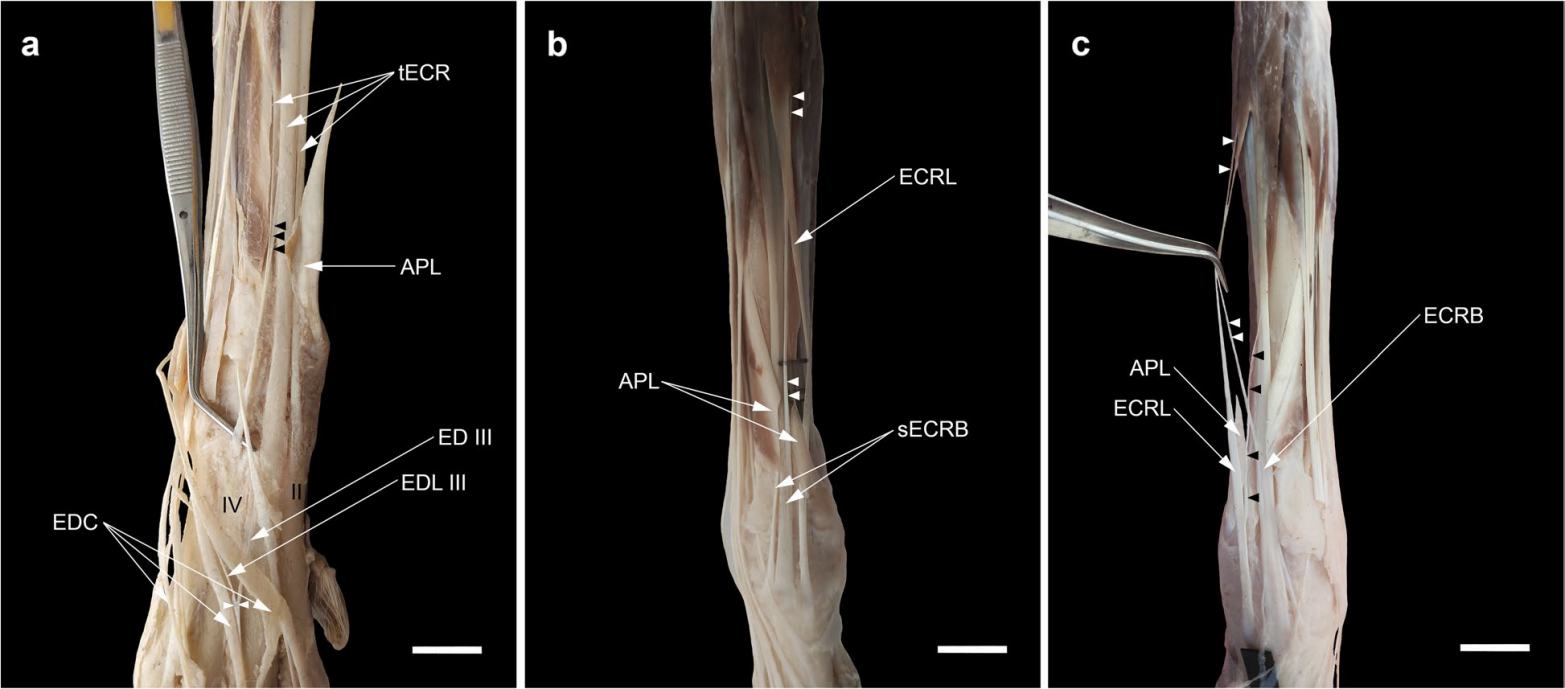
(**a**) The three tendons of extensor carpi radialis (tECR) with one extra tendon inserting on the base of the fourth metacarpal bone (IV) in the right antebrachium of the specimen No. 1; Black arrowheads, the fine tendon fibers from the main tendon to the extra one; white arrowheads, the site where the tendon of the extensor digiti I et II (ED III) joining that of the extensor digitorum lateralis (EDL III) to contribute to the extensor expansion of digit III. (**b** and **c**) Cross-connections of the tendons of the ECR of the right and left antebrachia of a female crossbred dog (No. 2). White arrowheads, an extra tendon arising from the medial belly of the ECR to join distally the ECRB; black arrowheads, an accessory tendinous slip connecting the ECRB to the ECRL on the left side (**c**). sECRB, a split tendon of ECRB to the base of the metacarpal bones III and IV (Type A of a split tendon of ECRB). Scale bar: 20 mm

In the right thoracic limb of a male specimen (1.5%), three tendons arising from the ECR muscle to the bases of the second, third, and fourth metacarpal bones were observed (Fig. 1a). In both thoracic limbs of a female specimen, the medial belly of the ECR muscle terminated in two tendons: one main tendon for the base of the second metacarpal bone (ECRL) and one accessory tendon joining the tendon of its counterpart (ECRB) near the distal end. Inversely, in the left thoracic limb of this specimen, the ECRL received an accessory tendinous slip from the ECRB (Fig. 1b, c).

### Splitting of the ECRL or the ECRB into two or three slips

It was noted that one of the ECR tendons split into two or three slips in 11 (16.2%) of the 68 thoracic limbs dissected. Eight (11.8%) of them belonged to the ECRB and three (4.4%) of them belonged to the ECRL. The division of the tendons occurred above the level of the carpus, at the carpus, or just proximal to the insertions into the bases of the metacarpal bones.

**Fig. 2:**
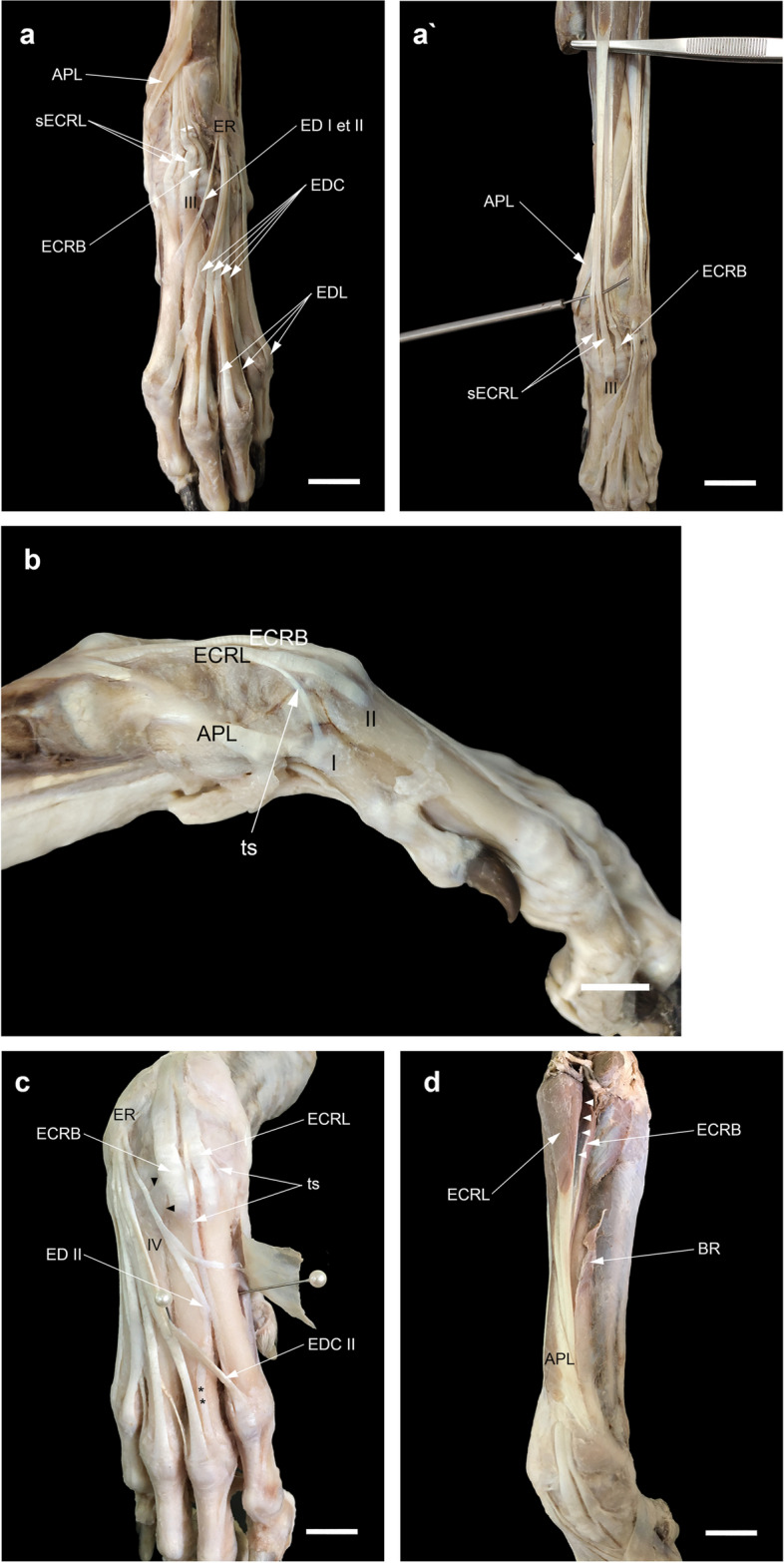
The split tendon of the ECRL (sECRL) found in this study. **a** A left manus (No.3) with sECRL which abaxial tendinous slip enclosing in a common tendon sheath (arrowheads) with the ECRB tendon to insert on the base of the third (III) metacarpal bone; (a') dissection of the same limb (**a**) by following the tendinous slips proximally; ER, extensor retinaculum. **b** A tendinous slip (ts) from the ECRL of the left manus (No. 4) to the base of the first (I) metacarpal bone. **c** A right manus (No. 5) with two extra tendinous slips (ts) from the ECRL, one short inserting on the base of the second (II) metacarpal bone, and one long extending more distally to contribute to the EDC to digit II (EDC II); the tendon of ED I et II to digit II (ED II) having a lateral deviation to merge with the tendinous slip (double asterisks) arising from the EDC II to digit III; black arrowheads, a triangular ligamentous structure arising from the ECRB tendon and spanning to the fourth (IV) metacarpal bone. **d** Dissection of the ECR of the same limb (**c**) with two completely separated bellies (note the arrowheads); BR, m. brachioradialis. Scale bar: 20 mm

In the initial dissection on a male specimen, the ECRB bilaterally appeared to have a double tendinous slip with the insertions into the base of the third metacarpal bone (Fig. 2a). However, by following the tendinous slips proximally, we found that one of the slips (abaxial) on the left side belonged to the ECRL and was enclosed at the carpus in a common synovial tendon sheath next to the ECRB tendon (Fig. 2a'). There were two specimens in which the ECRL at the carpus gave off an additional tendinous slip for the base of the first metacarpal bone on the left of one of them (Fig. 2b) and for the base of the second metacarpal bone on the right of the other (Fig. 2c). In addition, in the latter thoracic limb, the tendinous slip emanating from the tendon and extending to digit II was also classified in the subdivision of the long tendinous slip from the ECRL or the ECRB to the digits. The muscle bellies of the ECR were more developed and were entirely isolated from each other in this limb (Fig. 2d).

**Fig. 3:**
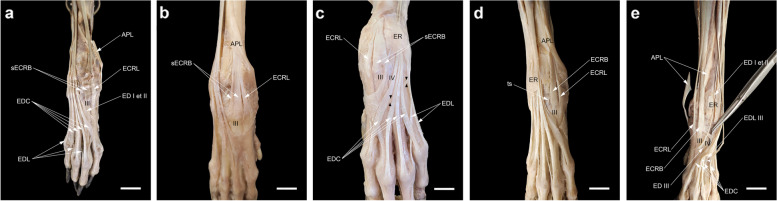
The split tendon of the ECRB (sECRB) found in this study (No. 3 (R), 6 (R), 2, 7, 8 (R & L)). **a**-**d** Types A to D of the sECRB based on the degree of separation. The absence of the EDL tendon of digit III on the left side of a female specimen (No. 7) (**c**) and its replacement by an oblique fascial band (arrowheads) should be noted. ts, a fine tendinous slip from the lateral margin of the ECRB, which inserted on the carpal joint capsule; III, third metacarpal bone; IV, fourth metacarpal bone. **e** A triangular ligamentous structure arising from the ECRB tendon and spanning to the fifth metacarpal bone in a left manus (No. 1) (held by tissue forceps). Scale bar: 20 mm

Four types of the ECRB were identified depending on the degree of separation. In Type A, the ECRB fully split into two slips both of which inserted into the base of the third metacarpal bone (one right thoracic limb already mentioned with the ECRL) (Fig. 3a) or into the bases of the third and fourth metacarpal bones (one bilateral) (Fig. 1b). In one case (bilateral), the ECRB partially split into two slips (Type B) which inserted into the base of the third metacarpal bone (Fig. 3b). In another one (bilateral), it split into two slips only at the distal end (Type C) and the abaxial slip inserted into the bases of both the third and fourth metacarpal bones (Fig. 3c). In one right manus, a fine tendinous slip split off at the carpus from the lateral margin of the ECRB (Type D) and inserted into the joint capsule proximal to the base of the third metacarpal bone (Fig. 3d).

In two manus (2.9%), a fan-shaped accessory tendinous expansion (with a ligamentous structure) diverged from the distal end of the ECRB to insert into the dorsal aspect of the base of the fourth metacarpal bone on the right side of one specimen (Fig. 2c) or after crossing the base of the fourth metacarpal bone to insert into the dorsomedial aspects of the shaft of the fifth metacarpal bone, distal to the base, on the left side of the other (Fig. 3e).

### A long tendinous slip from the ECRB or the ECRL to the digits (five manus -7.4%-)

**Fig. 4:**
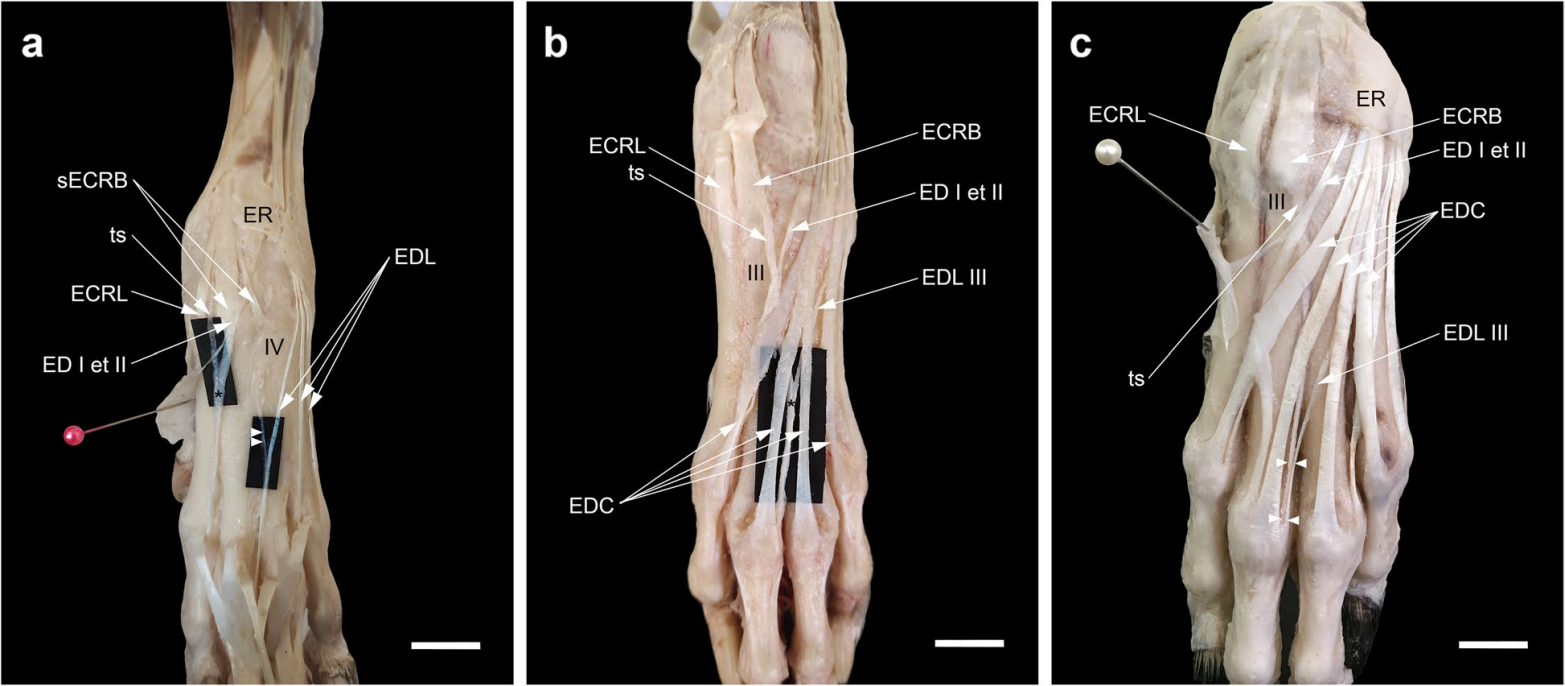
Dissections showing a long tendinous slip (ts) from the ECRB with the formation of a common tendon (*) with the tendon of ED I et II to digit II (No. 2 (L) (**a**) and the EDL tendon to digit III (EDL III) (No. 9, 10 (L) (**b**) or without formation of a common tendon with the latter tendon (arrowheads) (No. 5 (R & L) (**c**). Double arrowheads in (**a**), a tendinous slip joining the EDL III arose independently from the deep metacarpal fascia. Scale bar: 20 mm

The long tendinous slips of the ECRB or ECRL formed a common trunk with the other extensor tendons of the digits, i.e. digit II tendon of the extensor digiti I et II (ED II) (one left) (Fig. 4a) or EDL III (two manus, both left) (Fig. 4b) before joining the EDC tendon of the corresponding digit at the MCP joint or independently extending distally to join the latter tendon (one bilateral) (Figs. 2c, 4c).

### The contribution of tendon(s) arising from the EDL and/or ED I et II to digit III

**Fig. 5:**
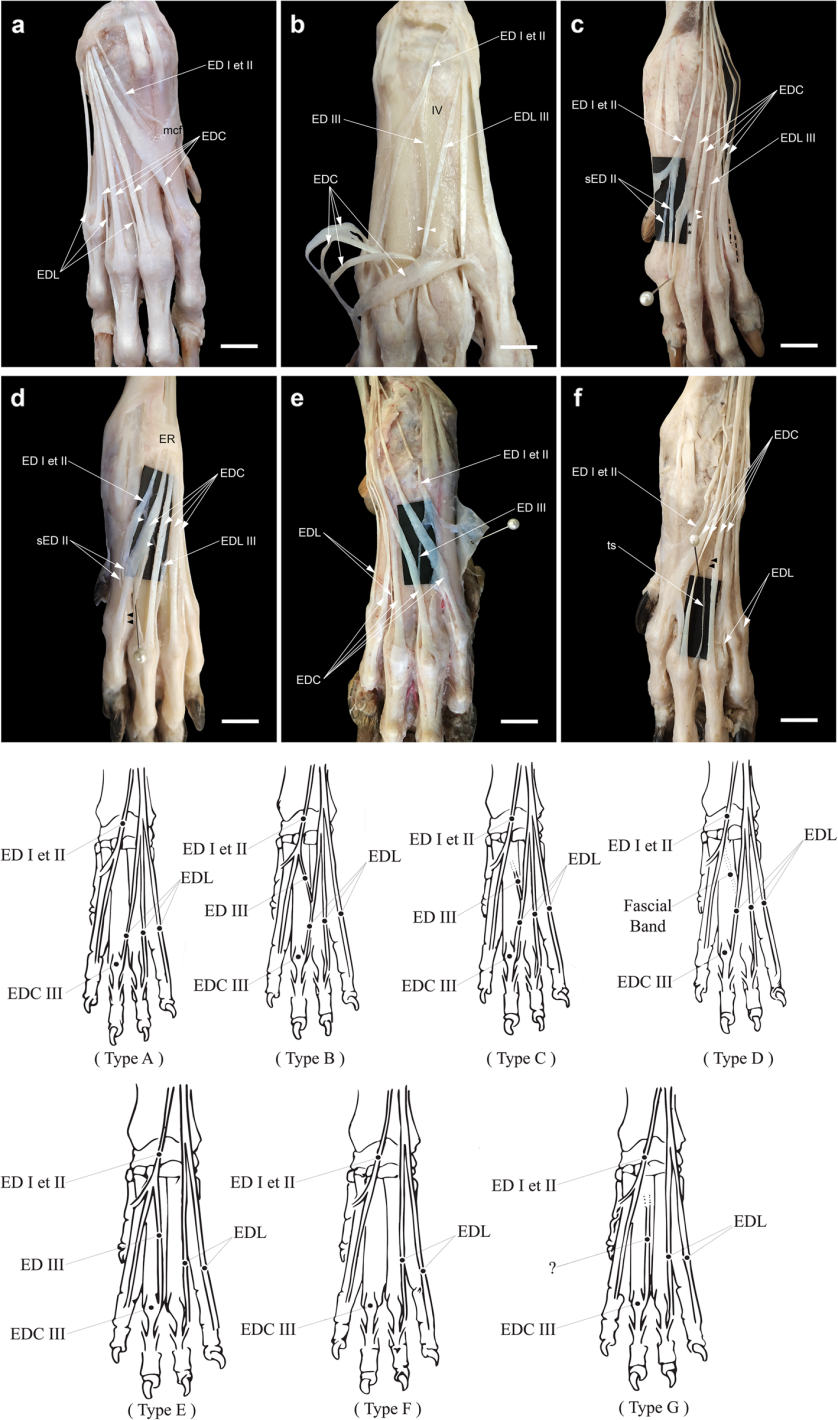
Dissections and illustration of variations of the contribution of tendon(s) arising from EDL and (or) ED I et II to digit III in this study. **a** Type A, (**b**) Type B, (**c**) (No. 2, 11 L) Type C, (**d**) (No. 12L) Type D, (**e**) (No. 11R) Type E, (**f**) (No. 7R & L) Type F, and (**g**) (No. 13L) Type G. Note a split ED II (sED II) with two insertions in (**c**) (No. 11L, 14R & L) and one insertion (black arrowheads) in (**d**) (No. 12L). Scale bar: 20 mm

The contribution of the tendon(s) arising from the EDL and/or ED I et II to digit III has been classified into several types as follows (Fig. 5):

- Type A: The tendon branch to digit III coming off from the EDL without the contribution of ED I et II was the most common pattern in our observations (67.6%) (Fig. 5a).
- Type B: The third branch of ED I et II joining EDL III was present in twelve manus (one on the left, three on the right, and four on both sides, -17.6%-) (Fig. 5b).
- Type C: In two limbs (3%), only the distal part of ED III joined EDL III, while its proximal part was not formed (Figs. 4a, 5c).
- Type D: In only one limb (1.5%), ED III joining EDL III was substituted by a fascial band (Fig. 5d).
- Type E: The absence of the tendon branch of the EDL to digit III (EDL III) was associated with the supply of an additional tendon branch of ED I et II to that digit (ED III) in only one limb (1.5%) (Fig. 5e).
- Type F: The absence of a tendon branch to digit III arising from these two muscles was found on both sides of one specimen (3%). EDL III was substituted on the left side by an oblique fascial band which crossed deep to the tendons of the EDC to reach the mid-shaft of the third metacarpal where it faded over the fascia (Fig. 3c).
- Type G: In one limb (1.5%), there was a tendon branch to digit III with its proximal part missing (Fig. 5f).

### Splitting of the second tendon of the ED I et II into two slips

The second tendon of ED I *et* II was found to have two slips and two insertions in three manus (one left and one bilateral) (Fig. 5c) as well as two slips and one insertion in one left manus (Fig. 5d).

### Splitting of the EDC tendon of digit III (EDC III)

In three manus (two left and one right -4.4%-), there was unilaterally a longitudinal fissure in the EDC III tendon at the MCP joint (Fig. S1).

### A fascial to tendinous accessory slip stemming from the extensor tendons of the digits

**Fig. 6:**
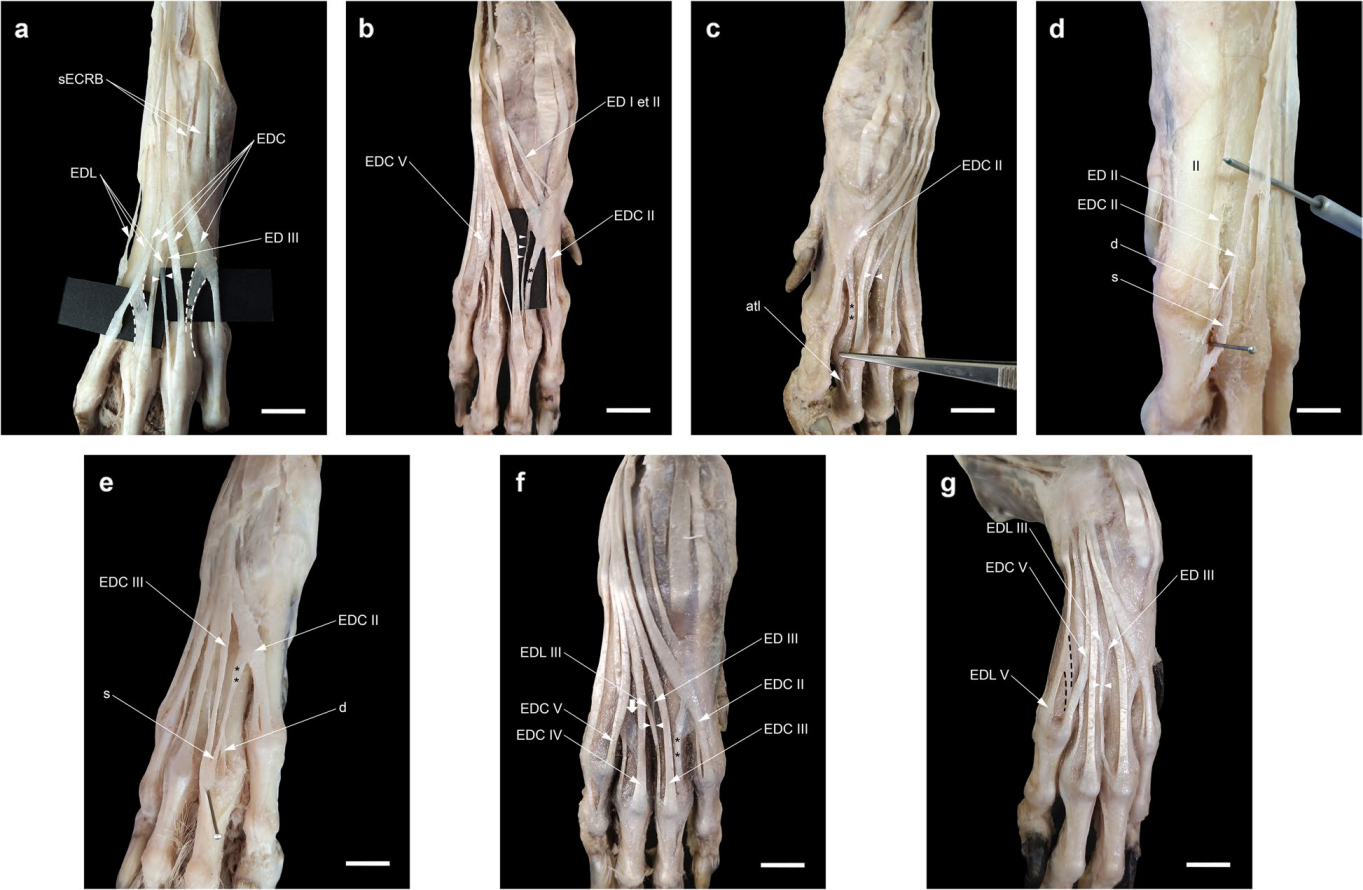
Dissections of the accessory (fascial to tendinous) slips stemming from the extensor tendons (EDC II, EDC V, and EDL V). The accessory slip from EDC II (double asterisks) is thinner than the main tendon (**a**) or the same thickness (**b**), and inserting into the MCP joint of digit III (**a**, **b**) or the axial tendon of m. interosseous (atI) (**c**). Note the divergent course of the EDC II and V proximal to the corresponding MCP joint in (**a**). The EDC II (**d**) or its accessory tendinous slip (**e**) having two superficial (s) and deep (**d**) slips. **f** (No. 15R) An oblique filamentous band (thick arrow) between the EDC tendons of digits IV and V in the fourth metacarpal space. (**g**) The accessory slip (black dotted borders) from the EDL to digit V (EDL V) inserting into the MCP joint of the same digit in three manus (one left (No. 11) and one bilateral (No. 16). Scale bar: 20 mm

In almost all cases, about two-thirds of the way down the metacarpus, the EDC tendon to digit II (EDC II) gave off a tendinous slip to digit III to insert into the joint capsule of the third MCP joint next to or deep to EDC III and exceptionally into the axial interosseous tendon of digit III. The tendinous slip was thinner than the main tendon or had the same thickness (Fig. 6a-c). There were two specimens in which EDC II (one left) or its tendinous slip (one right) near the MCP joint in turn split into two superficial and deep slips (Fig. 6d, e). In the former case, the superficial slip continued as a main tendon to the distal phalanx of digit II and the deep one was attached to the base of the proximal phalanx. In the latter case, the superficial slip united with EDC III at the MCP joint and the deep one inserted into the joint capsule. In only one case (on the right side), there was an oblique filamentous band which connected the EDC tendons of digits IV and V in the fourth metacarpal space (Fig. 6f). In three manus (one left and one bilateral), a fascial or tendinous slip (which was thinner than the base tendon) near the fifth MCP joint stemmed from the EDL to digit V (EDL V) to insert into the same joint capsule deep to the EDC to digit V (EDC V) (Figs. 5c, 6g). A fascial slip occasionally thickened by tendinous tissue arose from EDC V to attach to the capsule of the fourth MCP joint deep to EDC IV. This was observed in twenty-two forepaws (nine left, seven right, and three bilateral -32.3%-).

### The relationship of the attachment sites of the EDL, EDC, and interosseous tendons

**Fig. 7:**
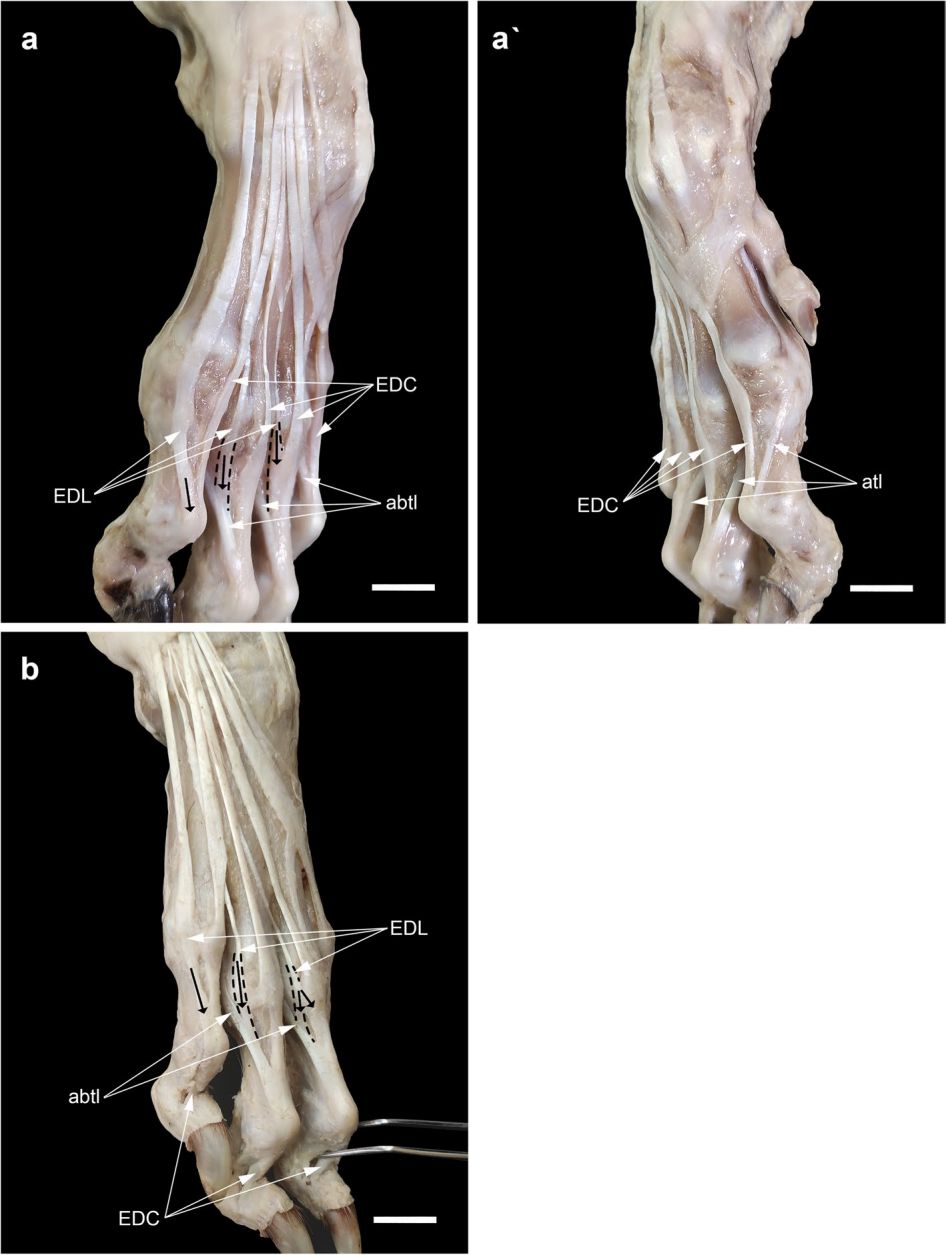
Dissections of the attachment sites of EDL (black arrows) in relation to the EDC and interosseous tendons. The absence of the abaxial tendon of m. interosseous (abtI) to EDC V in dorsolateral views (**a**, **b**) and the presence of the axial tendon of m. interosseous (atI) to EDC II in dorsomedial view (a') should be noted. Scale bar: 20 mm

In none of our dissections, the EDC tendon to digit V received the tendon of the interosseous muscle from the abaxial side. However, from both axial and abaxial sides, this muscle contributed to the EDC tendons serving the other digits (Fig. 7a, a’). Each of the EDL tendons inserted into its target tendon at different levels: the tendon to digit V passed dorsolateral to the MCP joint. Then, lateral to the digit, it united with the EDC V tendon at more distal levels on the proximal interphalangeal (PIP) joint (Fig. 7a). The tendon to digit IV united with the abaxial tendon of the interosseous muscle on the proximal phalanx. Finally, the attachment of the tendon to digit III with the EDC tendon and rarely with the abaxial tendon of the interosseous muscle occurred at the higher level on the MCP joint or the proximal phalanx of that digit (Fig. 7b).

### The relationship of the tendon insertion of the APL and the flexor retinaculum

**Fig. 8:**
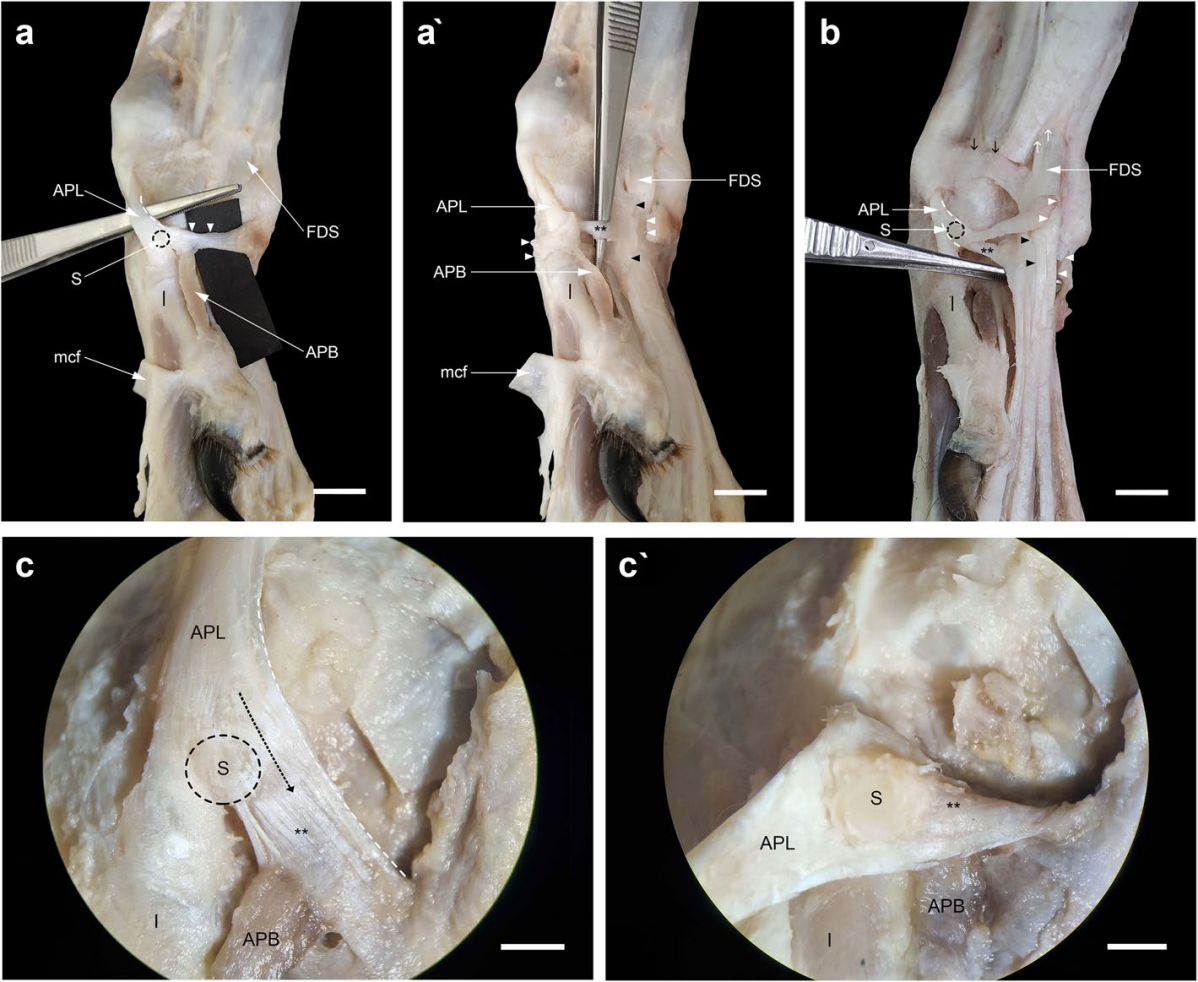
Medial views of the carpal region of the dog showing the insertion tendon of the abductor pollicis longus (APL) in relation to the flexor retinaculum. **a** Dotted circle, sesamoid bone (S); white arrowheads, superficial part of the flexor retinaculum; FDS, flexor digitorum superficialis; APB, abductor pollicis brevis; I, first metacarpal; mcf, reflected metacarpal fascia. the same limb after the transection of the superficial part of the flexor retinaculum. double asterisks, a tendinous like band deep to the superficial part of the flexor retinaculum extending from the sesamoid bone to the peritendineum (black arrowheads) of the FDS. **b** Other dissection of the carpal region for more emphasis. (↓), proximal band of the superficial part of the flexor retinaculum; (↑), the distal end of the antebrachial fascia. **c** Higher magnification of medial view of the carpal region under the stereomicroscope. Note some tendinous fibers of the APL (dotted arrow) extending proximal to the "S" to the FDS. (c') the tendon of APL is cut and reflected distad to show the precise site of the "S". Scale bar: 5 mm

Once the superficial layer of the flexor retinaculum was carefully incised from proximal to distal and reflected medially, a deeply situated oblique tendinous band extending from the sesamoid bone of the APL to the synovial sheath of the flexor digitorum superficialis (FDS) tendon could be defined (Fig. 8a, a', b). Indeed, some oblique tendinous fibers of the APL proximal to the sesamoid bone shifted to the FDS tendon (Fig. 8c). It seemed that part of the APL tendon inserted into the sesamoid and then continued with this tendinous band. The sesamoid bone was located at the angle formed by the two parts of the APL or at the palmar margin of the main part of the tendon going to the base of the first metacarpal bone (Fig. 8c', b). In the limbs where it was present, the abductor pollicis brevis (APB) was mainly attached either to the superficial layer of the flexor retinaculum or to the tendinous band rather than to the sesamoid bone.

**Fig. 9:**
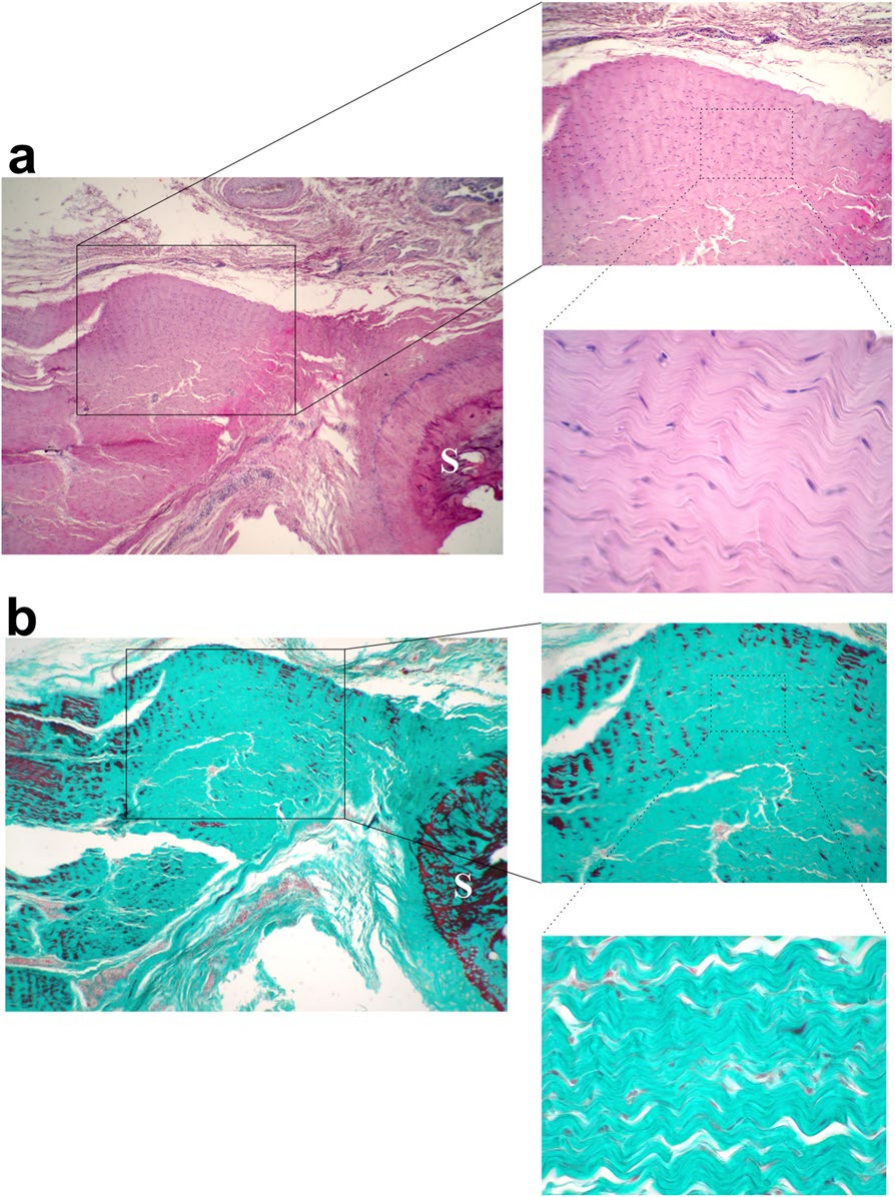
H&E (**a**) and Masson's trichrome (**b**) staining of longitudinal sections of the part of the APL tendon extending to the FDS. The collagen fibers were arranged in an undulating pattern with parallel orientation. Tenocytes of the tendon were routinely flattened (spindle-shaped), and nuclei arranged in rows between the collagen bundles. S refers to sesamoid bone

H&E and Masson's trichrome staining of longitudinal sections of the APL tendon revealed parallel orientation of the collagen fibers confirming the tendinous-tissue nature of the part that continued to the FDS tendon. Furthermore, tenocytes of tendon were routinely flattened, spindle-shaped, and nuclei arranged in rows between the collagen bundles (Fig. 9a, b).

### The morphometry of the parameters

In Table 2, all the measurements are summarized by means, standard deviations, and ranges. As shown in Table 3, although the mean width and length of each parameter were greater in male specimens than in female ones, they were statistically significant only in the following cases: the length of the forearm, the width of EDC IV at the MTJ, the width of EDC II, III, and IV at the insertion site, the width of ED II at the midmetacarpal, the length of EDC II, III, and IV, the length of EDL V, the length of the ECRL, the length of ED I, and the length of BR (*P* < 0.05). There was no significant difference between the values of the left and right thoracic limbs (*P* < 0.05) (Table 3).

**Table 2:** Dimensions of the anatomic structures in 60 distal forelimbs of adult crossbred dogs

Parameters	Range	Mean	Std. Deviation
Antebrachium length (cm)	15.8 -24.8	21.46	2.05
Manus length (cm)	10.1 -14.8	12.59	1.23
ECRL width MTJ (mm)	2.85 -9.05	5.46	1.12
ECRB width MTJ (mm)	2.88 -7.27	4.64	0.74
ECRL width insertion (mm)	2.4 -5.48	3.81	0.64
ECRB width insertion (mm)	3.39 -8.61	5.46	1.07
APL MTJ (mm)	4.3 -9.58	6.89	1.38
APL insertion to MC I (mm)	2.11 -4.94	3.53	0.65
APL insertion to sesamoid bone (mm)	2.86 -9.27	5.38	1.07
EDC II width MTJ (mm)	1.67 -4.97	3.37	0.74
EDC III width MTJ (mm)	1.36 -6.64	3.87	0.98
EDC IV width MTJ (mm)	1.43 -5.26	3.25	0.87
EDC V width MTJ (mm)	1.16 -3.38	1.90	0.42
EDC II width insertion (mm)	2.48 -6.94	4.93	0.85
EDC III width insertion (mm)	2.97 -7.96	5.60	0.92
EDC IV width insertion (mm)	3.43 -6.9	5.44	0.80
EDC V width insertion (mm)	2.39 -6.54	4.60	0.87
EDC II width mid MC (mm)	3.01 -5.96	4.36	0.71
EDC III width mid MC (mm)	1.76 -3.58	2.56	0.39
EDC IV width mid MC (mm)	1.64 -3.16	2.27	0.32
EDC V width mid MC (mm)	1.83 -3.75	2.68	0.49
EDL (Lat. belly) width MTJ (mm)	0.83 -3.38	1.77	0.45
EDL (Med. belly) width MTJ (mm)	0.97 -3.04	1.58	0.40
EDL V mid MC (mm)	1.68 -3.93	2.97	0.50
EDL IV mid MC (mm)	1.04 -3.13	1.92	0.40
EDL III mid MC (mm)	0.35 -2.19	1.35	0.39
ED I II MTJ (mm)	0.6 -1.82	0.94	0.27
ED I II mid II (mm)	0.42 -3.07	1.63	0.43
ED I II mid I (mm)	0.57 -2.71	1.66	0.47
ECRL length (cm)	10.1 -19.4	13.79	1.78
ECRB length (cm)	6.6 -11.6	9.08	1.30
APL length (cm)	3.4 -6.7	4.78	0.67
EDC II length (cm)	15.5 -22.8	19.15	1.90
EDC III length (cm)	17.4 -28.3	24.25	2.42
EDC IV length (cm)	19.4 -29.8	25.47	2.46
EDC V length (cm)	16.4 -24.9	20.93	2.07
EDL V length (cm)	15.1 -23.7	19.54	2.03
EDL IV length (cm)	13.6 -22.2	18.11	1.88
EDL III length (cm)	4.6 -11.7	7.90	1.32
ED I II length I (cm)	5.4 -11.1	7.53	1.26
ED I II length II (cm)	6.7 -13.8	9.98	1.47
BR width MTJ (mm)	0.65 -4.63	2.38	1.11
BR width mid MC (mm)	1.91 -8.5	4.72	1.56
BR length insertion (mm)	15.2 -46.81	28.37	8.50
BR width insertion (mm)	2.52 -10.05	5.15	2.74
BR length (cm)	13.6 -22.4	19.78	2.20

*BR* m. brachioradialis

**Table 3:** Dimensions of the anatomic structures in 60 distal forelimbs of adult crossbred dogs according to gender and sides

Parameters	Gender			Side		
	Female	Male	*p*-value	Left	Right	p-value
Antebrachium length (cm)	20.57 (1.97)	22.28 (1.8)	0.002**	21.46 (2.07)	21.46 (2.07)	1.000
Manus length (cm)	12.26 (1.32)	12.92 (1.12)	0.051	12.61 (1.24)	12.57 (1.25)	0.891
ECRL width MTJ (mm)	5.08 (1.13)	5.78 (1.05)	0.022	5.65 (1.12)	5.27 (1.1)	0.205
ECRB width MTJ (mm)	4.54 (0.84)	4.73 (0.66)	0.357	4.66 (0.59)	4.62 (0.87)	0.825
ECRL width insertion (mm)	3.68 (0.54)	3.92(0.71)	0.163	3.92 (0.68)	3.7 (0.59)	0.209
ECRB width insertion (mm)	5.36 (1.03)	5.59 (1.12)	0.43	5.6 (0.96)	5.32 (1.16)	0.333
APL width MTJ (mm)	6.72 (1.37)	6.98 (1.43)	0.501	7.08 (1.34)	6.7 (1.41)	0.294
APL width insertion to MC I (mm)	3.34 (0.66)	3.69 (0.59)	0.044	3.63 (0.68)	3.43 (0.61)	0.234
APL width insertion to sesamoid bone (mm)	5.15 (1.29)	5.56 (0.81)	0.168	5.22 (0.92)	5.53 (1.19)	0.265
EDC II width MTJ (mm)	3.17 (0.83)	3.56 (0.66)	0.061	3.35 (0.8)	3.4 (0.71)	0.807
EDC III width MTJ (mm)	3.88 (1.11)	3.87 (0.89)	0.987	3.71 (0.96)	4.02 [1]	0.235
EDC IV width MTJ (mm)	2.99 (0.86)	3.55 (0.79)	0.016*	3.23 (0.9)	3.27 (0.86)	0.839
EDC V width MTJ (mm)	1.88 (0.31)	1.94 (0.51)	0.64	1.87 (0.46)	1.93 (0.4)	0.597
EDC II width insertion (mm)	4.65 (0.89)	5.26 (0.74)	0.013*	5.03 (0.98)	4.83 (0.71)	0.404
EDC III width insertion (mm)	5.33 (0.97)	5.96 (0.8)	0.02*	5.64 (0.98)	5.57 (0.88)	0.788
EDC IV width insertion (mm)	5.21(0.84)	5.73 (0.7)	0.028*	5.43 (0.86)	5.45 (0.75)	0.921
EDC V width insertion (mm)	4.36 (0.98)	4.85 (0.68)	0.06	4.66 (0.92)	4.53 (0.83)	0.629
EDC II width mid MC (mm)	4.31(0.65)	4.44 (0.73)	0.487	4.22 (0.76)	4.5 (0.64)	0.133
EDC III width mid MC (mm)	2.43(0.36)	2.67 (0.39)	0.022*	2.58 (0.37)	2.53 (0.41)	0.639
EDC IV width mid MC (mm)	2.21 (0.28)	2.36 (0.34)	0.087	2.33 (0.32)	2.22 (0.32)	0.229
EDC V width mid MC (mm)	2.75 (0.54)	2.59 (0.42)	0.234	2.78 (0.51)	2.58 (0.45)	0.116
EDL (Lat. belly) width MTJ (mm)	1.73 (0.54)	1.79 (0.37)	0.615	1.8 (0.49)	1.75 (0.42)	0.699
EDL (Med. belly) width MTJ (mm)	1.52 (0.36)	1.63 (0.44)	0.332	1.53 (0.35)	1.63 (0.45)	0.354
EDL V width mid MC (mm)	2.96 (0.33)	3 (0.63)	0.745	2.94 (0.53)	2.99 (0.47)	0.738
EDL IV width mid MC (mm)	1.89 (0.42)	1.97 (0.38)	0.47	1.99 (0.46)	1.85 (0.31)	0.175
EDL III width mid MC (mm)	1.26 (0.3)	1.42 (0.46)	0.149	1.34 (0.39)	1.36 (0.4)	0.84
ED I et II width MTJ (mm)	0.85 (0.18)	1 (0.28)	0.019	0.95 (0.31)	0.93 (0.23)	0.785
ED II width mid MC (mm)	1.47 (0.33)	1.79 (0.48)	0.007**	1.57 (0.5)	1.69 (0.35)	0.321
ED I width mid MC (mm)	1.79 (0.4)	1.54 (0.5)	0.088	1.56 (0.48)	1.75 (0.45)	0.203
ECRL length (cm)	12.99 (1.64)	14.49 (1.67)	0.001**	13.83 (1.93)	13.75 (1.65)	0.872
ECRB length (cm)	8.61 (1.23)	9.46 (1.3)	0.017	9.03 (1.36)	9.12 (1.26)	0.777
APL length (cm)	4.69 (0.57)	4.87 (0.77)	0.312	4.74 (0.62)	4.82 (0.73)	0.65
EDC II length (cm)	18.3 (1.86)	19.9 (1.7)	0.002**	19.1 (2.01)	19.2 (1.83)	0.845
EDC III length (cm)	23.42 (2.61)	24.92 (2.1)	0.021*	24.09 (2.56)	24.4 (2.31)	0.624
EDC IV length (cm)	24.6 (2.69)	26.21 (2.06)	0.016*	25.38 (2.46)	25.56 (2.5)	0.785
EDC V length (cm)	20.31 (2.12)	21.42 (1.96)	0.05	20.94 (2.16)	20.92 (2.02)	0.978
EDL V length (cm)	18.63 (2.03)	20.3 (1.76)	0.002**	19.38 (2.03)	19.7 (2.06)	0.559
EDL IV length (cm)	17.75 (2.02)	18.46 (1.77)	0.171	17.99 (1.8)	18.22 (1.98)	0.641
EDL III length (cm)	7.43 (1.22)	8.37 (1.28)	0.011*	7.77 (1.23)	8.02 (1.41)	0.506
ED I II length I (cm)	6.91 (0.73)	7.99 (1.41)	0.003**	7.56 (1.34)	7.5 (1.2)	0.857
ED I II length II (cm)	9.65 (0.81)	10.24 (1.91)	0.156	9.85 (1.4)	10.11 (1.55)	0.527
BR width MTJ (mm)	2.1 (1.09)	2.73 (1.08)	0.104	2.52 (1.15)	2.2 (1.07)	0.408
BR width mid MC (mm)	4.41 (1.14)	5.08 (1.94)	0.228	4.73 (1.79)	4.7 (1.3)	0.967
BR length insertion (mm)	29.94 (8.15)	26.58 (8.82)	0.271	28.03 (8.99)	28.75 (8.21)	0.815
BR width insertion (mm)	3.27 (1.06)	7.03 (2.64)	0.084	5.13 (4.26)	5.18 (0.73)	0.986
BR length (cm)	19.08 (2.12)	20.61 (2.06)	0.039*	19.72 (2.41)	19.86 (2)	0.856

Significant *p*-value was < 0.05. *, **–statistical significance

## Discussion

### Variations of the ECR

Among the tendons of the extensors of the carpus and digits, the ECR showed striking variations. Phylogenetically, this muscle among tetrapods is derived from a common extensor mass of the humero-radial sector along with the brachioradialis and possibly the supinator [28, 29]. The progressively increasing separation of the ECR leads to the formation of two independent muscles (the extensor carpi radialis longus and brevis), promoting more precise functions for them. This condition is found in several mammal taxa such as Primates, most Rodentia, Talpidae, Tupaiidae, and Cynocephalidae [17, 30]. In the order Carnivora, varying degrees of fusion (less in felids and more in canids) to a complete separation (as in some procyonids) could occur in both parts of the ECR [31–39]. In this study, the complete division of the ECR resulted in the formation of two distinct and more developed muscle bellies and tendons in the right antebrachium of a male specimen (Fig. 2d), presenting a configuration similar to that described in the procyonids kinkajou (*Potos flavus*) and ring‐tailed coati (*Nasua nasua*), the hyaenid striped hyena (*Hyaena hyaena*)*,* some felids such as the domestic cat (*Felis catus*) and the lion (*Panthera leo*)*,* and variably in the mustelid lesser grison (*Galictis cuja*) and in the ailurid red panda (*Ailurus fulgens*) [5, 6, 10, 32, 36, 38–42]. Functionally, the complete separation of these two muscles promotes the actions of the carpus such as the adduction and the extension, which are especially important in arboreal species [32, 43]. When two ECRs exist as distinct structures, several connections may variably persist between their tendons, as described in the lion (*P. leo*) [42]. In the present study, it was noted that the additional tendon arising from the muscle bellies of the ECR in 3 (4.4%) of 68 limbs inserted into the base of the fourth metacarpal bone (one right) or joined the main tendon of its counterpart near the distal end (one bilateral). In a French text on comparative anatomy, it is briefly mentioned that the tendons of the ECR are connected by a strong intermediate tendon in the striped hyaena (*Hyaena hyaena*) and the brown bear (*Ursus arctos*) [16]. A similar tendinous connection has also been described in the ocelot (*Leopardus pardalis*) in which the proximal parts of the muscle are fused together. However, such connections have not been reported in the domestic dog [44, 45]. In addition, the literature on animal anatomy lacks a similar case in which an accessory tendon originates from the lateral belly of the ECR and inserts into the fourth metacarpal bone. This accessory tendon could potentially cause the additional extension of the carpus.

The ECRB tends to be bifurcated more than the ECRL with a frequency of 11.6% versus 4.4% in our specimens. Our study also found various bifurcation types of the tendons of the ECR based on the separation degree and the insertion sites on the other metacarpal bones. The tendinous slip of the ECRL inserted into either the second (one right limb that was additionally associated with a long tendinous slip to digit II) or the third (one left limb) and the first metacarpal bones (one left limb in a puppy). Among other carnivorans, a similar variation in the division of the ECRL has been recorded in a male Pampas fox (*Lycalopex gymnocercus*) [35], an otter (*Lutra* spp) with both tendinous slips inserting into the second metacarpal bone [46], and in a harbor seal (*Phoca vitulina*) with a tendinous slip inserting into the first metacarpal bone [16].

Based on the evidence of human anatomy and from the evolutionary point of view, the division of the ECRL inserting into the second and first metacarpal bones can occur from more distal levels (as a tendinous slip diverging from the main insertion tendon) to higher levels in the antebrachium (as a supernumerary belly inserting into the first metacarpal bone) [29, 47, 48]. The former condition found in higher, non-human primates as a common variation resembles that found in our dissections in the left limb of a puppy (Fig. 2b) [49, 50].

The ECRB was also noted to be partially or totally divided into two medial and lateral slips at different levels of the limb with the lateral one inserting variably into the third (one bilateral and one right limb), fourth (one bilateral), and both the third and fourth (one bilateral) metacarpal bones. Similarly, the lateral slip from the split tendon of the ECRB has been reported to insert on the fourth metacarpal bone in a dog specimen [16] and on the third metacarpal bone itself on both sides of a female Pampas fox specimen [51]. This shows that the tendon insertion of the ECRB has a distinct tendency to migrate laterally. Lastly, since the migration of the insertion of the ECR from the carpus to the metacarpus occurs over the course of evolution, the insertion of a fine slip from the ECRB tendon onto the carpus detected in a right limb dissected in this study is likely a reversion to a primitive condition (Fig. 3d) as it also inserts on the carpus in amphibians and reptiles [29, 52, 53]. Furthermore, a long tendinous slip extending from the metacarpal attachment of the ECRL and ECRB towards the digits was present in one (1.5%) and four (5.9%) of the 68 limbs dissected, respectively. Among these, the tendinous slip from the ECRB insertion tendon to digit III forming a common trunk with EDL III (found in 2 limbs) has been mentioned as a common variation in an illustration in Miller’s Anatomy [7]. Ziegler also found a tendon arising from the insertion point of the ECR to digit III [54]. However, an anomalous long tendinous slip from the ECRL and ECRB (associated with a split tendon) contributing to the tendon to digit II in two other limbs has not been reported heretofore in the anatomy literature. Based on the results of this study, the high tendency of the ECR tendon to send a tendinous slip to the digits may be considered a phylogenetic trait indicating a transformation in the evolution of its tendon of insertion more distally from the metacarpal to the digits. This will probably be manifested with a higher frequency in future generations of canids. Based on this study, in two manus (Figs. 2c and 4a), these tendinous slips are detached as a part of the split tendons of the ECR before the main tendons attach onto the bases of the metacarpal bones. Thus, at least in these two cases, the tendinous slips could be functionally active and contribute to the transmission of muscle force distally towards the digits for additional extension.

In two of the 68 manus, a triangular ligamentous structure was found arising from the distal end of the ECRB with its apex at the third metacarpal. It spanned to the fourth metacarpal bone (one right) or the fifth metacarpal bone (one left) which could be phylogenetically considered as an example of the formation of a new ligament (bone-to-bone attachment) from a tendon. It may be a homologue to what has been reported in human hand as the first intermetacarpal ligament from the insertion tendon of the ECRL at its distal end to the first metacarpal bone [50].

### The variations of the contribution of tendon(s) arising from EDL and (or) ED I et II to digit III

**Fig. 10:**
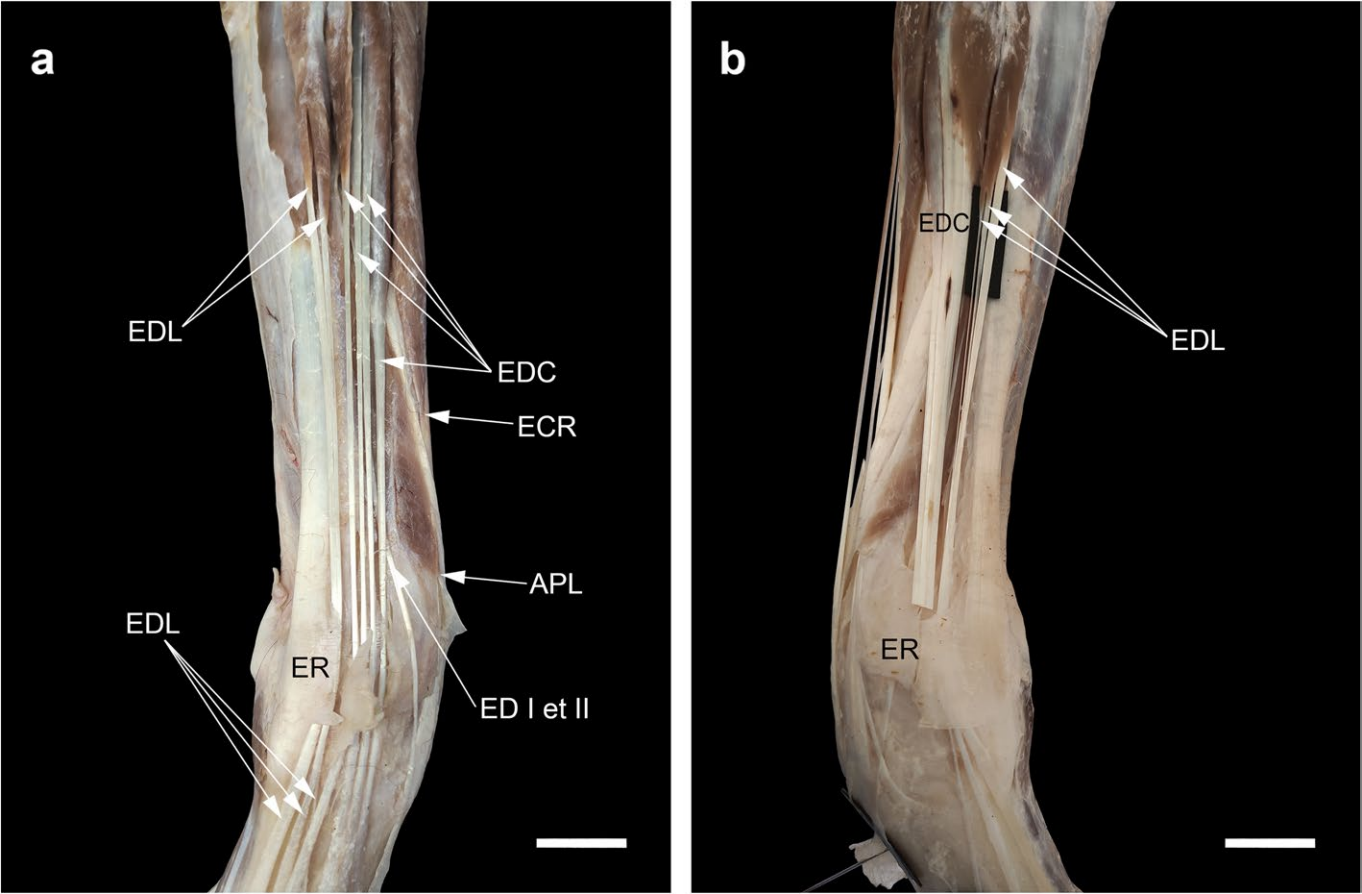
Dissection of the extensor digitorum lateralis (EDL) of domestic dogs in this study. **a** In normal conditions, the EDL has a double tendon, medial and lateral, in which the medial one split into two unequal slips after passing through extensor retinaculum (ER). **b** Dissection of a variant in which the EDL having a triple tendon at the myotendinous junction (MTJ) found in the left antebrachium of a female dog. Scale bar: 20 mm

The number of the tendons of the EDL muscle to the lateral digits varies among carnivores including three tendons to digits III-V in the domestic dog and up to four tendons to digit II-V in the domestic cat (*F. catus*), the ocelot (*L. pardalis*), the tiger (*P. tigris*), the lion (*P. leo*), and the jaguar (*P. onca*) [6, 19, 37, 42, 44]. In standard veterinary anatomy texts, the EDL muscle in the domestic dog is shown to have two bellies with terminations in two tendons (the larger one to digit V and the smaller one to digit IV). After the tendons diverge from each other, the smaller tendon also gives off a tendon branch to digit III at the carpus (Fig. 10a) [6, 7, 10]. In only one dissection in this study, however, the tendon serving digit III originated from the medial belly at the MTJ (Fig. 10b). In three manus (one right and one bilateral) dissected in this study, the EDL tendon to digit III (ED III) was not developed similar to that described in the golden jackal (*Canis aureus*), the African wild dog (*Lycaon pictus*), the dhole (*Cuon alpinus*), the red fox (*Vulpes vulpes),* the striped hyena (*Hyaena hyaena*), and the spotted hyena (*Crocuta crocuta*) [4, 31, 34, 41, 55]. Furthermore, we found that the third branch of ED I et II joining EDL III (Type B) was more common (17.6%) than that mentioned in Miller’s *Anatomy* as a rare variation [7]. This type is also variably seen in canids such as the crab-eating fox (*Cerdocyon thous*) and *L. gymnocercus* [35, 56] and commonly in felids such as the domestic cat (*F. catus*) and the tiger (*P. tigris*) [6, 37, 57]. Regarding Type G of the contribution to digit III, in Miller’s *Anatomy* [7], citing the study of Ziegler [58], it is stated that in exceptional cases, the EDL has only two tendons for digits V and IV and the tendon to digit III independently originates from the fascia distal to the carpus. However, based on the several variations in relation to the EDL and ED I et II encountered in this study (especially Type E), why should not we consider the tendon branch to digit III of ectopic origin associated with ED I et II?

Embryologically, although the early development of the limb tendons occurs independently of the related muscles, the late tendon and muscle morphogenesis requires a reciprocal relationship between them [59, 60]. The tendon to digit III is described as a ‘slip or branch’ arising from the EDL or ED I et II without a distinct muscle belly. Therefore, from the loss of the proximal part of the tendon branch to its complete absence during morphogenesis might be linked to a lack of close interaction with its corresponding muscle belly.

### Variations of the BR

**Fig. 11:**
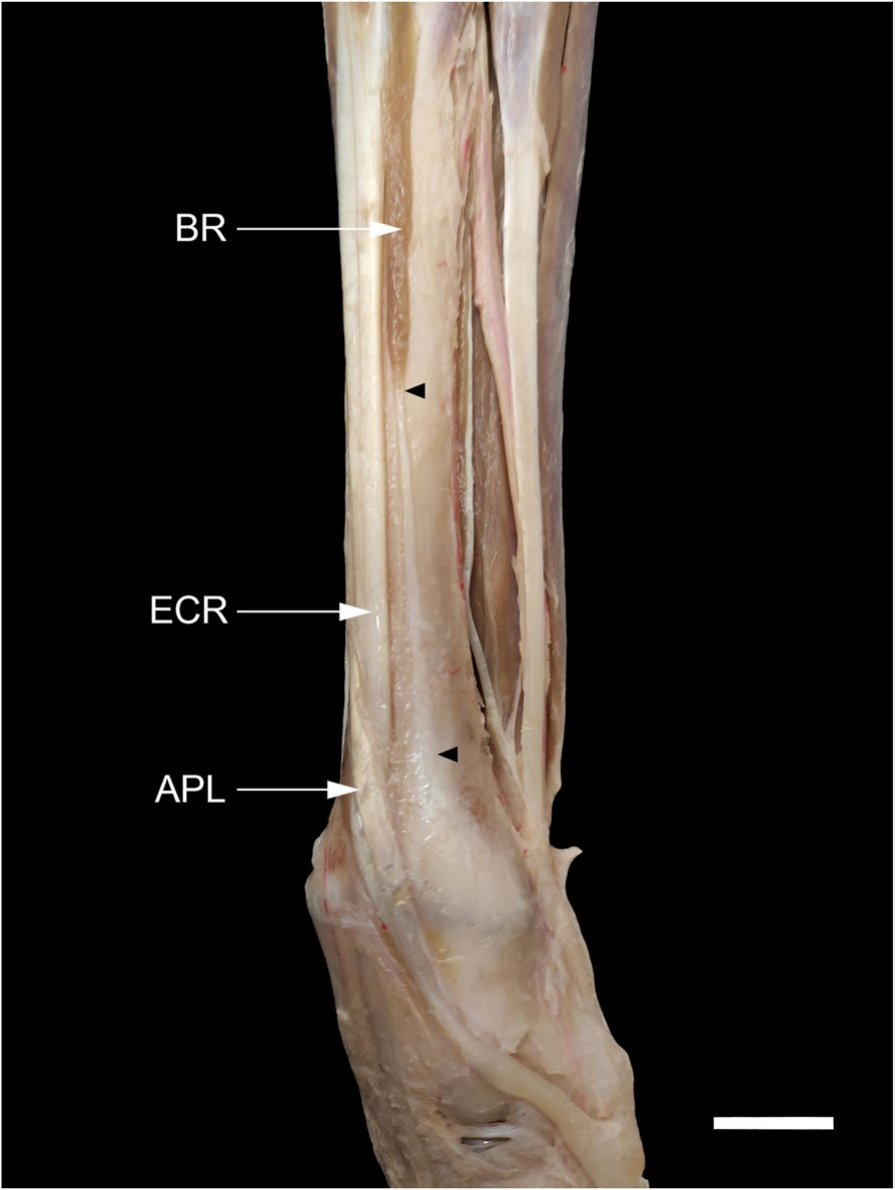
The craniomedial view of dissection of m. brachioradialis (BR) with a long and distinct tendon extending from the MTJ to near the radial styloid process (arrowheads). Scale bar: 20 mm

We noted the presence of the BR bilaterally and unilaterally respectively in 18 (53%) and 5 (14.7%) of the 34 dogs dissected compared with 40 (40%) bilateral and 15 (15%) unilateral of the 100 dogs dissected by Pestana et al. [61], 110 (33.74%) bilateral and 65 (19.94%) unilateral of the 326 dogs dissected by Santos Junior et al. [62], and 35 (38.9%) of the 90 dogs dissected by Wakuri and Kano [63]. The insertion of the BR muscle was variably attached through a long and distinct tendon (Fig. 11), a thin aponeurosis, or a direct attachment of some muscle fibers to the bone periosteum of the radius, proximal to its styloid process. Among carnivorans, other additional insertion sites have also been reported as in *L. pardalis* to the flexor retinaculum and retinaculum holding the APL tendon, in *P. uncia* to the tendon of APL rather than the distal radius, and in *A. fulgens* to the pronator teres muscle [3, 38, 44]. In addition, another insertion site onto the proximal row of the carpus has been described in *L. pardalis* and *P. onca* [19] and onto the dorsal aspect of the radial carpal bone in the Anatolian bobcats (*Lynx lynx*) [64].

Regarding interspecific variations, BR is lacking or underdeveloped in carnivorans with cursorial activity such as Canidae and Hyaenidae as compared with Procyonidae, Mustelidae, Ursidae, Ailuridae, and Felidae in which the rotatory movement ability is more important [4, 19, 36, 38, 41, 44, 64–67].

### Functional anatomy of the fascial to tendinous slips stemming from the extensor digitorum tendons

Another finding of the present study is that the fascial to tendinous slips stemming from the extensor digitorum tendons insert into the adjacent digit (digit III, IV, or V). Except in one dissection, they were not arranged in the same way as the connecting band between adjacent tendons as observed in the human hand [68]. Hence, proving the functional anatomical significance of these structures in the domestic dog is somewhat difficult. Interestingly, they branched off from the extensor digitorum tendons which showed a slight divergence on their course proximal to the MCP joints (i.e. the EDC of digits II and V and rarely the EDL of digit V). For instance, the EDC to digit II had the maximum medial divergence from the midline of the manus. As a result, a tendinous slip to the MCP joint of digit III was present as thick as the base tendon in all specimens of this study. Therefore, they might merely help to hold the extensor digitorum tendons in place to prevent their sliding over the metacarpals or they may serve to more stabilize the MCP joints of the two longest digits (i.e. III and IV) which bear the greater weight of the thoracic limb. On the other hand, since the tendinous slip from the EDC of digit II was directly attached to the EDC of digit III at the MCP joint or even more distally to the axial tendon of the interosseous muscle in some specimens, it can be considered as a supplying tendon for digit III in the absence of a tendon branch from the EDL or ED I et II. Finally, in our dissections, the presence of an oblique connecting band between the EDC tendons of digits IV and V in the fourth intermetacarpal space was noted in only one limb (one right) (Fig. 6f). This band may be homologous with Type 1 Juncturae tendinum seen in the second intermetacarpal space of the human hand [11, 68].

### The attachment sites of the insertion tendons

According to the dissections in the present study, the sesamoid bone was located where the APL tendon was bifurcated into two branches. As already mentioned, it appeared that part of the APL tendon, after inserting on the small sesamoid bone, continued with a tendinous band deep to the superficial part of the flexor retinaculum to reach the peritendineum of FDS. More recently, González-Rellán et al. re-examined the anatomy of the palmar aspect of the carpus in the domestic dog [69]. They described the superficial part of the flexor retinaculum as having a distal margin extending from the sesamoid bone of the APL to the subcutaneous tissue of the palmar pad and passing over the FDS tendon. However, the description of this tendinous band dissected in our study is missing in their study. Our observations showed that this tendinous band, because of its appearance, should be considered as part of the insertion tendon of the APL rather than the flexor retinaculum. From the morphogenetic standpoint regarding the development of tendons, one hypothesis can be derived according to which since the tendons and skeletal components have a common mesodermal origin, in the absence or underdevelopment of a target bone, the tendon can connect to other tendons sometimes even to the fascia of another muscle [70, 71]. This can be generalized to the phylogenetic aspects of tendon evolution. Compared to other Caniformia such as some species of Ursidae, Ailuridae, and Procyonidae in which the radial sesamoid bone is well-developed and serves as a second site for the attachment of the insertion tendon of APL [38, 72–75], it has undergone a reduction in size during evolution in Canidae (including the domestic dog) because of their adaptability for ambulatory terrestrial locomotion. Therefore, at least in domestic dogs, a part of the insertion tendon of APL might have migrated to attach directly to the nearest tendon, i.e. the FDS tendon.

In their study, González-Rellán et al. also describe an oblique aponeurotic-like band as a continuation of the distal end of the antebrachial fascia from the palmar aspect of the carpus to the dorsomedial metacarpal region [69]. In addition to this observation, our dissections showed that, on its way to the metacarpals, this aponeurotic-like band attached to the capsule of the first MCP joint, sent a slip to that of the second MCP joint in some limbs, and finally faded over the tendon of the EDC to digit II. In most limbs dissected in this study, the tendon branch of ED I et II to digit I inserted into this aponeurotic-like band at the second metacarpal. Then, they were easily lifted from the surface of the metacarpal (Fig. 4a, c, 5e). Only in rare specimens of the present study, it continued to the head of the first metacarpal bone. Similarly, in some wild canids (such as *L. pictus*), it does not insert onto the vestigial digit I and is terminated on the second metacarpal bone [34]. Functionally, this might be because the underdeveloped digit I in domestic dogs does not require further extension. Regarding the interspecific variations, the second tendon of ED I *et* II with two axial and abaxial slips in 3 manus (4.4%) dissected in this study is similar to that reported in *C. thous* [76].

According to standard veterinary anatomy textbooks, each EDC tendon insertion receives reinforcing tendons on each side of the proximal phalanx from interosseous muscles [6, 7, 10]. In contrast, our dissections showed that the EDC tendon to digit V lacked the contribution of the tendon of the interosseous muscle from the abaxial side. Instead, the EDL tendon of the corresponding digit was united with it at the site of the PIP joint in the same direction. Although Popesko’s schematic illustration of the dog’s manus anatomy [77] is consistent with the dissections in this study, the axial tendon of the interosseous muscle for the EDC to digit II (found in our specimens) has been ignored in it.

### Morphometric analyses

In the present study, comparing the data of the length and width of each parameter in the two genders led to notable findings. The lengths of the antebrachium, some extensor tendons (such as the EDC tendons to the digits II, III, and IV), the EDL tendon to digit V, and ED I et II to digit I as well as the width of the EDC to digits II, III, and IV at the insertion sites were significantly greater in male specimens than in female specimens. This shows a sexual dimorphism in the evolution of these parameters in the thoracic limb of the domestic dog.

### Limitations

The statistical population of this study was relatively small (*n* = 68 thoracic limbs). Hence, more accurate and impressive results could be obtained in a higher population. An in-depth study of this work may expand the observations on more different breeds of dogs such as shepherd, hunting, and living room dogs. This approach could help explain whether the variations in the tendon anatomy of these muscles are related to different biomechanical stresses or not. The morphometry of the extensor tendons could be complemented by measuring their thickness. However, due to their very low thickness in some places or the non-uniformity of some of them at the MTJ site, this was not done in this study. Histological studies are needed to confirm whether the tendinous-like bands arising from some extensor tendons to the next digits are tendinous, ligamentous, or fascial.

## Conclusions

This research demonstrated that the interconnections of the two ECR tendons might exist through accessory tendinous slips in the domestic dog in which the proximal parts of the muscles are blended together. This point should be taken into consideration during dissection. The tendon insertions of the ECR have a tendency to split for other metacarpal bones (I and IV) or send a long tendinous slip to digits (the ECRL to digit II -1.5%- and the ECRB to digits II -1.5%- or III -4.4%-). This indicates that the insertion tendons of the ECR are still dynamic and tend to divide and migrate without involving the muscle bellies, probably to preserve the evolutionary adaptations related to the muscle function. This feature agrees with the hypothesis proposed by some authors based on which the evolutionary origin of the muscle and its tendon is single [78]. This is because the different types of separation in the present study show that the division begins from the more distal parts of the tendon (Type C) and extends to the proximal parts, forming accessory tendons. According to the reports in primate studies, this division even exists in its ultimate form as a separate supernumerary muscle [79]. In domestic dogs, the tendons serving digit III are marked by an increase or decrease in number. The rare intraspecific variations of the extensor tendons of the manus described in the present research are valuable not only clinically but also phylogenetically. The results could be compared with those of wild carnivoran species in future studies for a better understanding of the phylogenetic relationships in morphology and locomotor ability. Nevertheless, their functional importance requires more investigations.

## Methods

For this study, 68 thoracic limbs from 34 dogs of both sexes (16 females and 18 males) were examined. This study was approved by the Animal Ethical Committee of the School of Veterinary Medicine, Shiraz University, Shiraz, Iran. 12 adult crossbred dogs were obtained from the kennel of the School of Veterinary Medicine, Shiraz University, for the purpose of teaching veterinary anatomy during two years, 4 dogs (including a German Shepherd, a Doberman Pinscher, and two crossbred dogs) which had the surgical intervention of the abdominal cavity and were candidates for euthanasia for reasons other than orthopedic problems were taken from the Department of Veterinary Surgery, and the remaining 18 crossbred dogs (including 2 puppies) were found dead on the highways of Shiraz in the Southwest of Iran. The thoracic limbs of the specimens found dead were severed from above the elbow and preserved in a 10% formaldehyde solution. The rest of the specimens were humanely euthanized with an intravenous injection of sodium pentobarbital (85 mg/kg) followed by exsanguination from the cannulated common carotid artery. After the confirmation of their death, the cadavers were perfused with a standard fixative solution containing 10% formaldehyde through the same artery and stored at 4–6 ºC for at least three weeks until dissection. A caudal incision was made through the skin of the antebrachium and manus from the point of the elbow distad around the carpal and metacarpal pads to the interdigital space of digits III and IV. Then, the muscles of the craniolateral compartment including the BR and the extensors of the carpus and digits were cleaned and identified. The ECU was excluded since it does not function only as an extensor. To better observe the extensor tendons of the carpus and digits, the extensor retinaculum was longitudinally incised. By considering the possible variations, all tendons of each muscle were carefully isolated by cutting the synovial sheath. Then, they were traced from the myotendinous junction (MTJ) to their insertions onto the bases of the metacarpal bones or phalanges. Two or more divisions of a tendon distal to the MTJ were defined as tendinous slips. The variations in the tendons, tendinous slips, and the connections between them were classified and described in detail. The Nomina Anatomica Veterinaria (ICVGAN, 2017) 6th edition was used for most anatomical terms [9].

### Histological studies

For this purpose, APL tendons of randomly selected thoracic limbs were removed from near the sesamoid bone to attachments to the base of the first metacarpal bone and the flexor digitorum superficialis tendon (FDS), and they were fixed in 10% formalin. Then, tendon samples were dehydrated in increasing concentrations of ethanol solution (70, 80, 96, 96, 100, 100%), rinsed with xylene and embedded in paraffin. The paraffin blocks were sectioned in 5–6 μm thickness and stained with haematoxylin and eosin (H&E), and Masson's trichrome. Histological slides were observed using a light microscope (Olympus CX-21) equipped with a digital camera (True Chrome II).

### Morphometry

Two adult dogs (from German Shepherd and Doberman Pinscher breeds) and two puppies were excluded from the morphometric measurements. In total, 60 thoracic limbs from adult crossbred dogs of both sexes (14 females and 16 males) were measured. The following measurements including the length (using a thread and then placing it on a steel engineering ruler) and/or width (using a 0.01 mm digital caliper) of each structure were recorded. Each measurement was performed twice for more accuracy:

- The length of the antebrachium from the most proximal part of olecranon to the styloid process of the ulna.
- The length of the manus from the styloid process of the ulna to the distal ends of digits III and IV.
- The length of each tendon from the MTJ to its insertion.
- The length of each tendinous slip from the point of separation of a base tendon to the point of union with other extensor tendons or the tendinous slips of the digits.
- The width of each tendon at the MTJ and insertion (on the bases of the metacarpal bones and the extensor expansions of the distal phalanges) levels as well as the width of the extensor tendons and tendinous slips of the digits at the midmetacarpal level.

The dimensions of the BR, if present, include its total length from the lateral supracondylar crest of the humerus to near the styloid process of the radius, the length of its tendon insertion, the width of the muscle belly in the middle of the antebrachium, and the width of its tendon insertion at the beginning and the end.

### Statistical analysis

All measured data followed a normal distribution based on the Kolmogorov–Smirnov test. Therefore, the independent samples t-test was used to investigate and compare the mean values obtained from measuring the tendons of the left and right thoracic limbs of the dogs as well as to compare the parameters between male and female specimens. The analysis was performed by the SPSS software (version: 26). *P* < 0.05 was considered significant.

### Supplementary Information

**Additional file 1:** **Fig. S1.** Dissection of the EDC III tendon having a longitudinal fissure at the MCP joint in one right (a) and two left (b and c) manus. Scale bar: 20 mm.

## Data Availability

The datasets generated and/or analyzed during the current study are available from the corresponding author on request.

## Abbreviations

APB: Abductor pollicis brevis
APL: Abductor pollicis longus
abf: Antebrachial fascia
atI: Axial tendon of m. interosseous
abtI: Abaxial tendon of m. interosseous
ECRB: Extensor carpi radialis brevis
ECRL: Extensor carpi radialis longus
ECU: Extensor carpi ulnaris
ED I et II: Extensor digiti I et II
EDC: Extensor digitorum communis
EDL: Extensor digitorum lateralis
ER: Extensor retinaculum
FDS: Flexor digitorum superficialis
mcf: Metacarpal fascia
MCP: Metacarpophalangeal joint
MTJ: Myotendinous junction
ts: Tendinous slip
