# Supplementary material for: Intraspecific anatomical variations of the extensor tendons of the carpus and digits with a reexamination of their insertion sites in the domestic dog (Canis lupus familiaris): a cadaveric study
Source: BMC Vet Res. 2023 Oct 9;19:197. doi: 10.1186/s12917-023-03750-w (PMC10561507; doi:10.1186/s12917-023-03750-w)
Supplement: Supplementary file 1 — Additional file 1: Fig. S1. Dissection of the EDC III tendon having a longitudinal fissure at the MCP joint in one right (a) and two left (b and c) manus. Scale bar: 20 mm. [file 12917_2023_3750_MOESM1_ESM.docx]

**Supplementary Material**

**
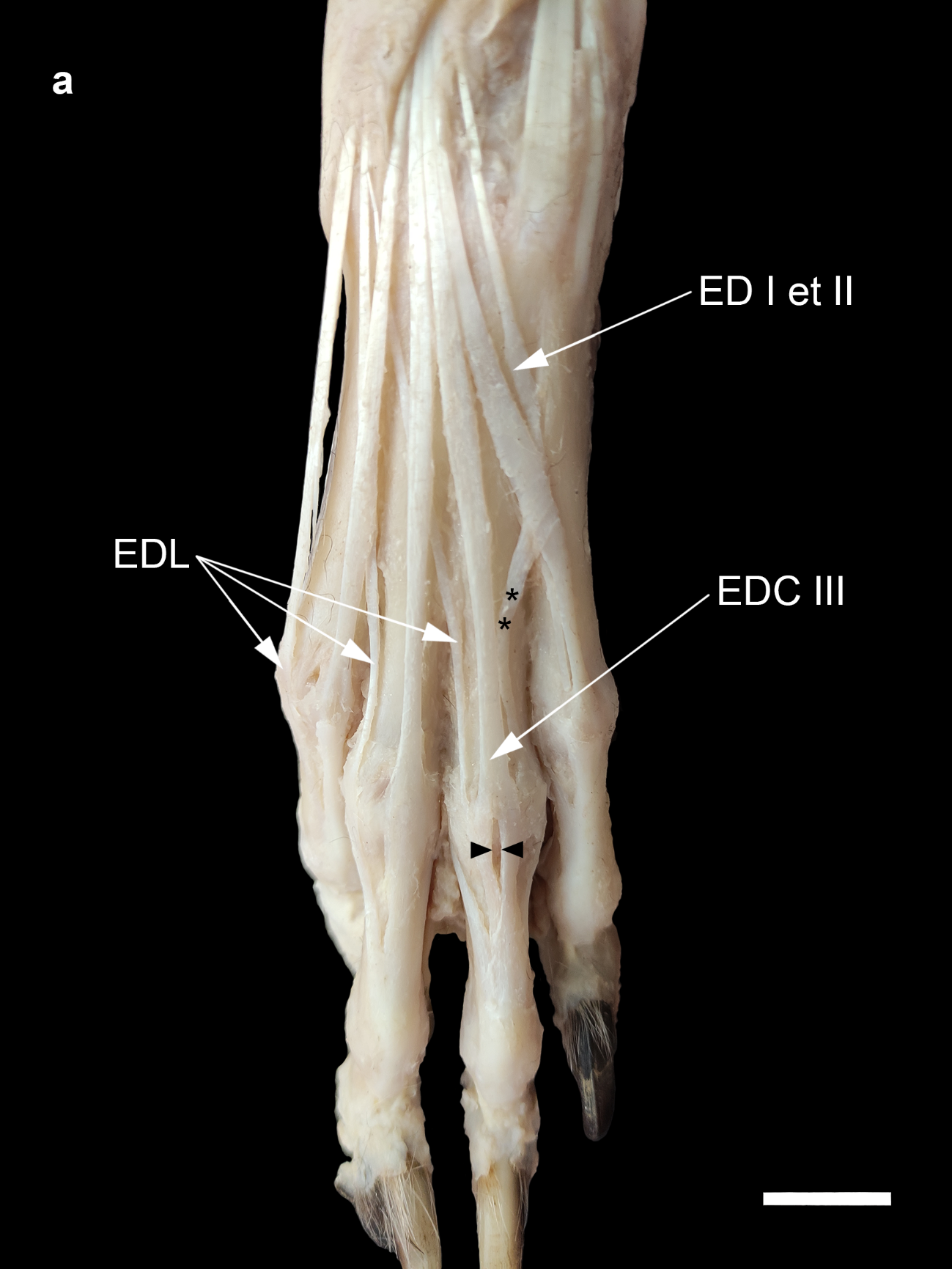

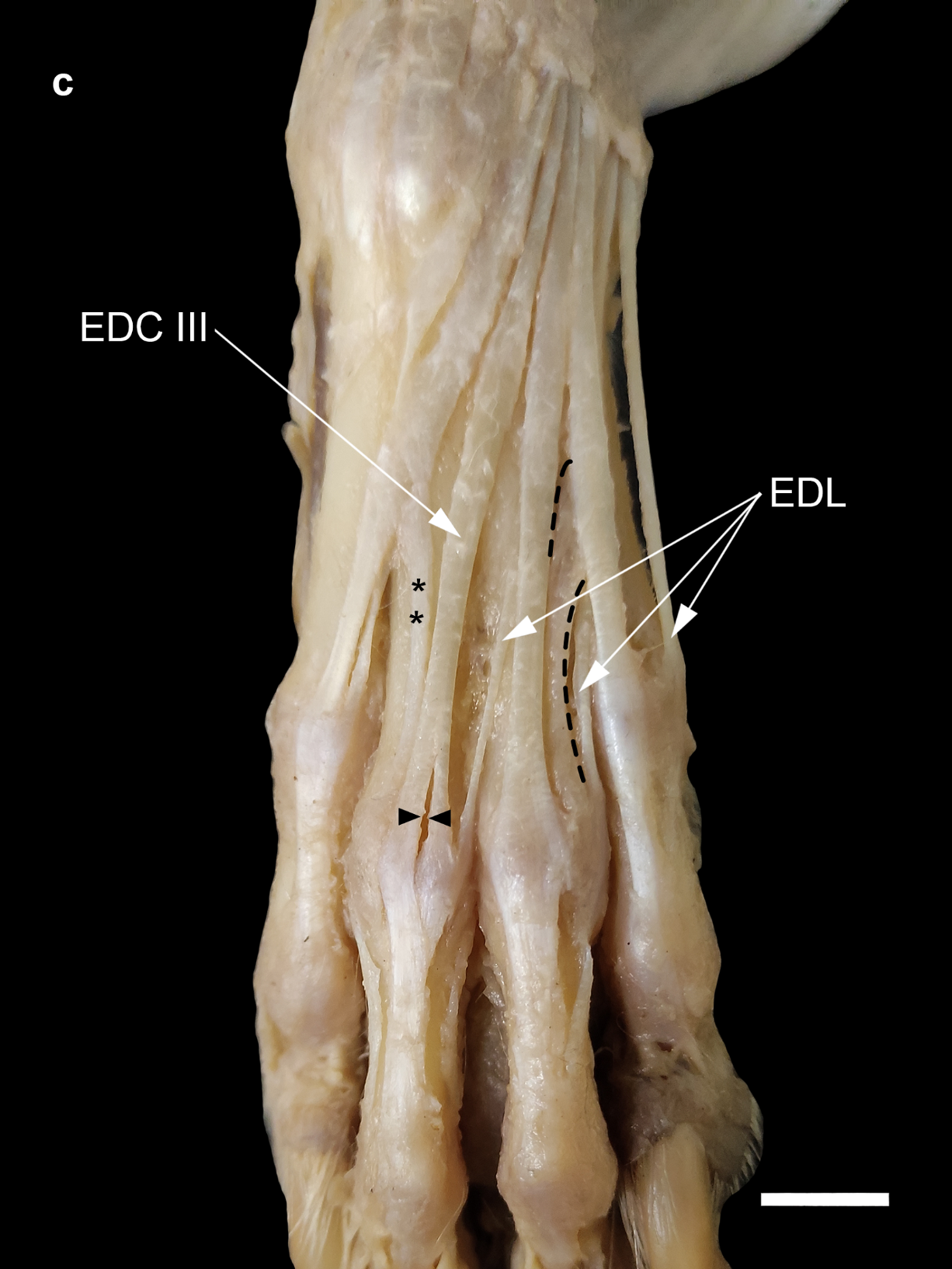

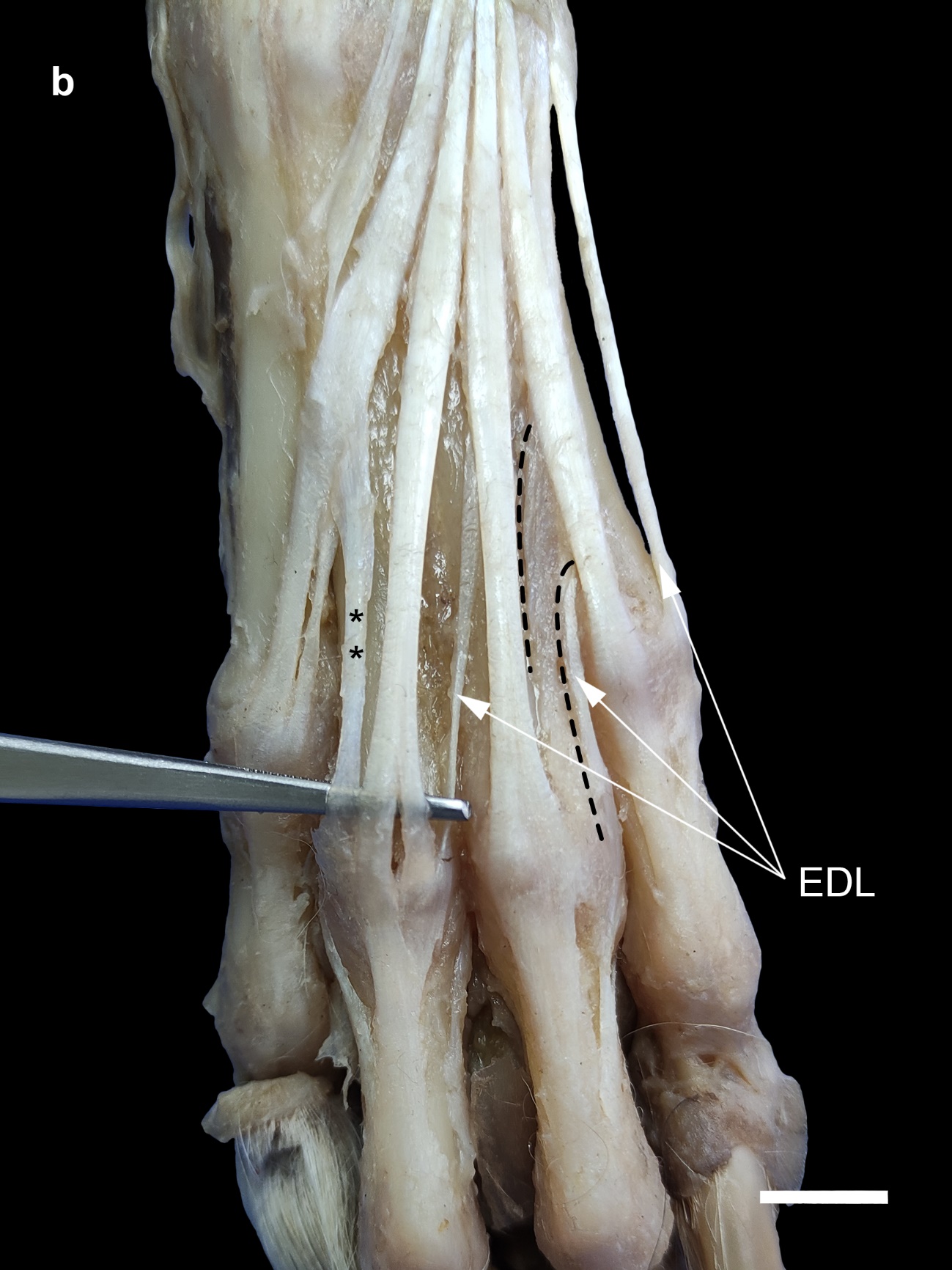
**

**Fig. S1** Dissection of the EDC III tendon having a longitudinal fissure at the MCP joint in one right (a) and two left (b and c) manus. Scale bar: 20 mm.
